# Timescales of influenza A/H3N2 antibody dynamics

**DOI:** 10.1371/journal.pbio.2004974

**Published:** 2018-08-20

**Authors:** Adam J. Kucharski, Justin Lessler, Derek A. T. Cummings, Steven Riley

**Affiliations:** 1 Centre for the Mathematical Modelling of Infectious Diseases, London School of Hygiene & Tropical Medicine, London, United Kingdom; 2 Department of Epidemiology, Johns Hopkins Bloomberg School of Public Health, Baltimore, Maryland, United States of America; 3 Emerging Pathogens Institute, University of Florida, Gainesville, Florida, United States of America; 4 MRC Centre for Outbreak Analysis and Modelling, Department of Infectious Disease Epidemiology, School of Public Health, Imperial College London, London, United Kingdom; Weatherall Institute of Molecular Medicine, University of Oxford, United States of America

## Abstract

Human immunity influences the evolution and impact of influenza strains. Because individuals are infected with multiple influenza strains during their lifetime, and each virus can generate a cross-reactive antibody response, it is challenging to quantify the processes that shape observed immune responses or to reliably detect recent infection from serological samples. Using a Bayesian model of antibody dynamics at multiple timescales, we explain complex cross-reactive antibody landscapes by inferring participants’ histories of infection with serological data from cross-sectional and longitudinal studies of influenza A/H3N2 in southern China and Vietnam. We find that individual-level influenza antibody profiles can be explained by a short-lived, broadly cross-reactive response that decays within a year to leave a smaller long-term response acting against a narrower range of strains. We also demonstrate that accounting for dynamic immune responses alongside infection history can provide a more accurate alternative to traditional definitions of seroconversion for the estimation of infection attack rates. Our work provides a general model for quantifying aspects of influenza immunity acting at multiple timescales based on contemporary serological data and suggests a two-armed immune response to influenza infection consistent with competitive dynamics between B cell populations. This approach to analysing multiple timescales for antigenic responses could also be applied to other multistrain pathogens such as dengue and related flaviviruses.

## Author summary

It is challenging to determine the true extent of influenza infection and immunity within a population, because a person’s immune response to a specific influenza strain depends both on past infections with that strain as well as immunity generated by related influenza strains. To untangle these processes, we developed a mathematical model that considered individual histories of influenza infection and immune dynamics acting at multiple timescales. We combined this model with surveys of antibody levels in different individuals, showing how antibody dynamics are influenced by a short-lived, broadly cross-reactive response against a wide range of strains that wanes over time to leave a long-term response against a narrower collection of strains. By accounting for such short- and long-term responses, we also found that it was possible to obtain better estimates of the frequency of influenza infection. These methods could help to guide the design of studies to estimate key aspects of influenza immune dynamics or to estimate historical infection rates and would also be applicable to other pathogens with multiple strains.

## Introduction

Immunity against influenza A can influence the severity of disease [1, 2], the effectiveness of vaccination strategies [3], and the emergence of novel strains [4, 5]. Understanding the accumulation of immunity and infection has proven challenging because observed human antibody responses—typically measured by haemagglutination inhibition (HI) assays or microneutralisation titres [6]—reflect a combination of past infections to specific strains and the potentially cross-reactive responses generated by these infections [7].

Several aspects of influenza antibody dynamics have been well described through measurement of individual antibody repertoires. In particular, there is evidence of long-lived, strain-specific antibody responses directed against epitopes in the haemagglutinin (HA) glycoprotein head domain [8, 9], as well as weaker, cross-reactive responses directed at conserved epitopes in the HA stalk [7]. There is also evidence that influenza infection leads to ‘back-boosting', generating a transient, broadly cross-reactive response against historical strains [10–12]. In addition, it has been suggested that influenza responses are influenced by antigenic seniority, with strains seen earlier in life shaping subsequent antibody responses [13]. This is a refinement on the earlier concept of ‘original antigenic sin', whereby the largest antibody response is maintained against the first infection of a lifetime [14].

Although there are established techniques for the analysis of single-strain immunising pathogens such as measles [15], potential cross-reactivity between different influenza A strains means serological analysis must account for the dynamics of antibody responses across multiple infections [16]. The concept of an antibody landscape has been put forward as one way to represent the immune response developed as a result of a sequence of processes such as infection, antibody boosting, antibody waning, and cross-reactivity [10]. Previous work has also used cross-sectional data to explore the life course of immunity by explicitly modelling both the processes of infection and immunity [17]. However, such analysis could not quantitatively estimate the contribution of different antibody mechanisms operating at multiple timescales. These cross-reactive dynamics, combined with measurement error in available assays, have made it challenging to uncover an individual’s exposure history from serological responses. It has been shown that measurement error in HI assays can lead to uncertainty in the estimation of serological status [18], and cross-reactive antibody dynamics can make it difficult to estimate the true extent of influenza infection during an epidemic [2]. Accurate estimation of attack rates is crucial for estimating influenza burden and hence the design and evaluation of vaccination campaigns [19].

To quantify antibody kinetics over time and estimate historical infections with influenza A/H3N2, we used a dynamic model of immune responses that generated expected titres against specific strains [17] by combining infection history—which was specific for each individual—with an antibody response process that was universal across individuals. We assumed that the response included both a short-term and long-term component (S1 Fig). The short-term component consisted of a boost in log titre following infection, which decayed over time, as well as a rise in log titre as a result of cross-reaction with antigenically variable strains. The long-term response featured a boost in log titre, which did not decay, and a separate cross-reaction process that led to increased titres against other strains. Titres were also influenced by antigenic seniority, with later infections generating lower levels of homologous boosting than that generated against strains encountered earlier in life (see Materials and methods). Historical strains were assumed to follow a smooth path through a two-dimensional antigenic space over time [20] (S2 Fig). We fitted this model to 2 publicly available serological datasets in which participants were tested against a panel of A/H3N2 strains. The first contained cross-sectional HI and microneutralisation data for individuals living in the Guangdong province in southern China, collected in 2009 [13, 21, 22]; the second included longitudinal HI data from Ha Nam in Vietnam [23], with sera collected between 2007 and 2012 [10, 24].

## Results

**Fig 1:**
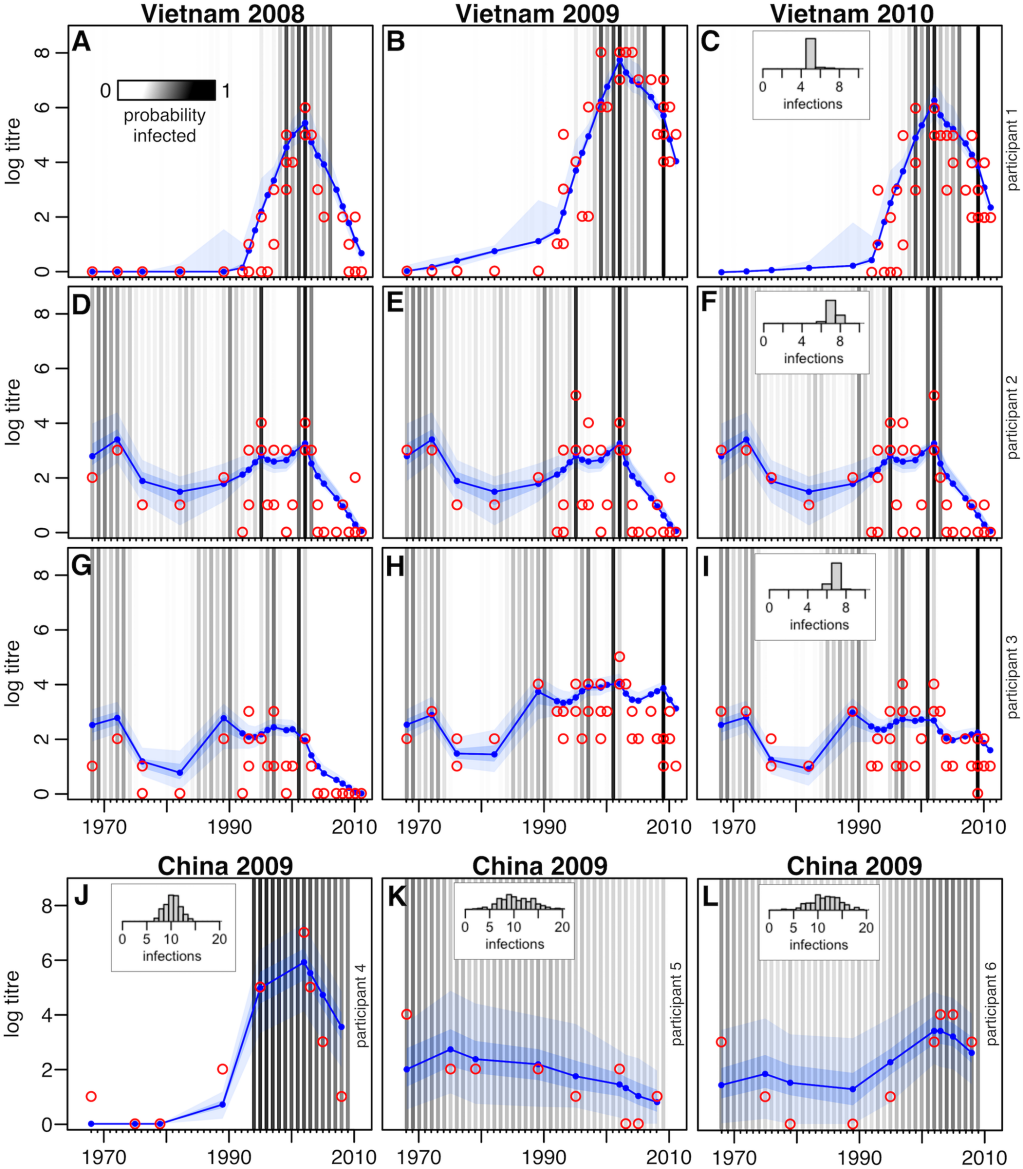
Representative individual-level HI titres against influenza in Vietnam (A–I) and southern China (J–L). (A–C) Example participant from Vietnam dataset. Strong evidence of infection in 2009, leading to rise in titres and back boost from broad short-term cross-reaction, then decay in following year. Red points show observed titre. Titres correspond to strains isolated in each year, based on antigenic path shown in S1 Fig. Blue lines show median titre in fitted model, with blue regions showing 50% and 95% MCMC credibility intervals. Black lines show samples from the posterior distribution of individual infection histories, with opacity indicating the probability of infection (i.e., proportion of MCMC samples that estimated infection in that year). The (C) inset shows distribution of estimated total infections. (D–F) Second Vietnam participant. No estimated infections between 2008 and 2010, so titres are at equilibrium. (G–I) Third Vietnam participant. Infection in 2009 leading to broad boost, with titres generally highest against recent strains (H) then decline to equilibrium, with lower mean titres against recent strains as a result of antigenic seniority (I). (J–L) Cross-sectional results from southern China, indicating (J) evidence of multiple recent infections for participant aged 15 years; (K) decline in titres as a result of antigenic seniority (participant aged 41 years); (L) evidence of infections early and late in life (participant aged 57 years). MCMC, Markov chain Monte Carlo.

Using our serological model, we jointly estimated influenza infection history for each study participant, as well as subsequent antibody response processes and assay measurement variability. Although the contributions of short- and long-term processes to antibody responses cannot be robustly estimated from cross-sectional data [17], simulation studies showed that both timescales were identifiable using a simulated dataset similar to that of the Vietnam samples (S3 and S4 Figs). We therefore included the short-term dynamic antibody processes in the model when fitting longitudinal data but not when fitting to cross-sectional data. The fitted model could reproduce both cross-sectional and longitudinal observed titres for each participant (Fig 1, Table 1), and it was possible to identify specific years with a high probability of infection and the corresponding antibody profile this infection history had generated (S5 and S6 Figs). Using the longitudinal Vietnam data, we could identify specific years in which individuals had a high probability of infection, particularly during the period of testing (Fig 1A–1I). There was more variability in estimates from the cross-sectional HI China data, although time periods with a high probability of infection could still be identified (Fig 1J–1L).

**Table 1:** Proportion of titres with model residuals less than 1, 2, and 3. Residuals are calculated as the absolute difference between median log titre estimated in the model and observed log titre, across all individuals and test strains.

Dataset	Residuals ≤ 1	Residuals ≤ 2	Residuals ≤ 3
China 2009 (microneut)	0.78	0.96	0.99
China 2009 (HI)	0.79	0.96	1.00
Vietnam 2007–2012 (HI)	0.72	0.90	0.97

Abbreviation: HI, haemagglutination inhibition.

The model fits to longitudinal data described an antibody response to influenza that is initially dominated by a broadly cross-reactive response, which rapidly decays, leaving a long-term response that cross-reacts only with antigenically similar viruses (Table 2, S7–S9 Figs). We estimated that primary infection generated a short-lived boost of an average of 2.69 (95% credibility interval [CrI]: 2.50–2.88) units of log titre against the infecting virus (a 4-fold rise would be equivalent to a 2-unit rise in log titre) and a long-term boost of 2.02 log-titre units (95% CrI: 1.96–2.08). The short-term response decayed quickly: we estimated that the response had reached its final equilibrium level after 1.27 years (95% CrI: 1.19–1.35). The timescale of this short-term response is consistent with previous qualitative estimates based on laboratory-confirmed infections, which suggested there was a negligible change in titre more than 1 year post infection [10, 11, 25].

**Table 2:** Parameter estimates for models fitted to HI and microneutralisation data from southern China and Vietnam. Median estimates are shown, with 95% credible intervals in parentheses.

Parameter	Vietnam 2007–2012 (HI)	China 2009 (HI)	China 2009 (microneut)
Long-term boost (*μ*_1_)	2.02 (1.96–2.08)	0.97 (0.85–1.13)	1.38 (1.14–1.66)
Short-term boost (*μ*_2_)	2.69 (2.50–2.88)	–	–
Long-term cross-reaction (*σ*_1_)	0.130 (0.128–0.132)	0.099 (0.084–0.114)	0.130 (0.106–0.146)
Short-term cross-reaction (*σ*_2_)	0.031 (0.026–0.035)	–	–
Observation error (*ε*)	1.29 (1.27–1.31)	1.50 (1.41–1.59)	1.69 (1.56–1.82)
Antigenic seniority (*τ*)	0.039 (0.035–0.042)	0.016 (0.012–0.021)	0.020 (0.015–0.027)
Waning (*ω*)	0.79 (0.74–0.84)	–	–

Abbreviation: HI, haemagglutination inhibition.

For the long-term response inferred from longitudinal data, we estimated that cross-reactivity between infecting strain and tested strain dropped off at a rate of 0.26 units of log titre (95% CrI: 0.25–0.27) per unit of antigenic distance between them. The estimated drop was larger than that inferred with the cross-sectional China HI data: log titres decreased by 0.096 (0.07–0.12) with each antigenic unit. This suggests the cross-sectional model may be capturing some of the broadly cross-reactive response, which was explicitly included in the model fitted to longitudinal data. As a sensitivity analysis, we also fitted the cross-sectional model independently to microneutralisation assay titres for the same individuals and test strains in the China study (Table 2). A previous study of these data found high correspondence between HI and microneutralisation titres [22]. Applying our model to these microneutralisation data, we estimated that cross-reactive log titres decreased by 0.18 (0.12–0.23) with each antigenic unit. The specific parameter estimates for boosting and cross-reactivity were different between the HI and microneutralisation assays, but the distribution of estimated number of infections in the population was similar, with a median of 15 infections (95% CrI: 4–30) using the HI data and 14 (2–29) using the microneutralisation data (S10 Fig). Moreover, the estimates for antigenic seniority and observation error were not significantly different between the two assays (Table 2). The estimated error structure of the two assays suggests that for a log titre mid-way between two integer cutoffs (e.g., 1.5), there was a 0.26 (0.25–0.28) probability that the microneutralisation test would return the correct log-titre measurement (i.e., 1) and a 0.23 (0.22–0.25) probability of a correct observation in the HI assay. For the broader short-term response, the model fitted to longitudinal HI data suggested cross-reactive titres decreased by 0.082 (95% CrI: 0.067–0.098) with each unit of antigenic distance. This result suggests that short-term titres are influenced by antigenic distance, albeit weakly, and hence provides quantitative support for previous suggestions that the observed broad short-lived boost is part of a memory B cell response [10].

**Fig 2:**
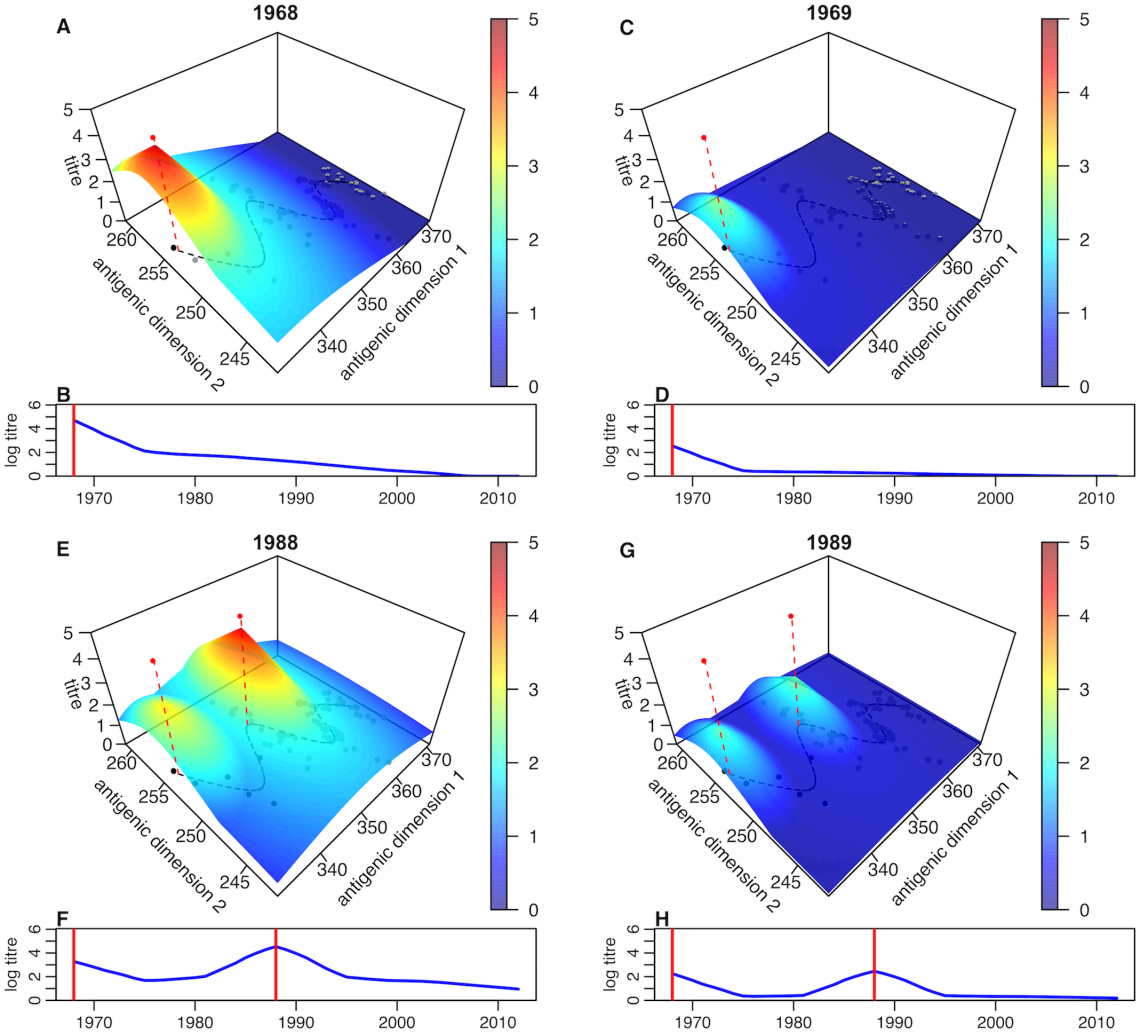
Expected titres against strains at different points in antigenic space for a given infection sequence. (A) Simulated log titres against different strains in antigenic space following a single infection in 1968, with test conducted in 1968. Parameters are drawn from the maximum a posteriori model estimate. Red vertical dashed line shows antigenic location of infecting strain. Black points at the base show location of strains isolated up to this year; grey points show location of strain isolates in subsequent years; black dashed line shows antigenic summary path used to fit model (S1 Fig). (B) Estimated titres along the antigenic summary path (dashed black line in (A)). Red line shows year of infection. (C) Simulated log titres following on single infection in 1968, with test conducted in 1969. (D) Estimated titres along antigenic summary path in 1969. (E) Simulated log titres following infections in 1968 and 1988, with test conducted in 1988. (F) Estimated titres along antigenic summary path in 1988. (G) Simulated log titres following infections in 1968 and 1988, with test conducted in 1989. (H) Estimated titres along antigenic summary path in 1989.

To illustrate the inferred short- and long-term antibody dynamics against A/H3N2, we used our infection history model to simulate antibody responses following 2 sequential infections, the first in 1968 and the second in 1988 (Fig 2). Following primary infection, individuals would be expected to have raised titres to strains in nearby regions of antigenic space, but these titres would quickly decay to leave a more localised long-term response (S11 Fig). Upon secondary infection, a similar boost in titres would be observed, which would not be present in tests conducted in subsequent years. This highlights the importance of accounting for multiple timescales when analysing immune assay data: in simulations, serology taken in 1988 indicated a rise in titre to the first infecting strain compared to serology between 1969 and 1987 and showed detectable titres against all strains in the region of antigenic space between the two infecting strains (Fig 2F). However, serology taken 1 year later only displayed localised responses against the infecting strains (Fig 2H). Depending on time of sampling, our results suggest it would be possible to observe either longitudinal increases or decreases in log titres against previously seen strains or stable log titres [7].

**Fig 3:**
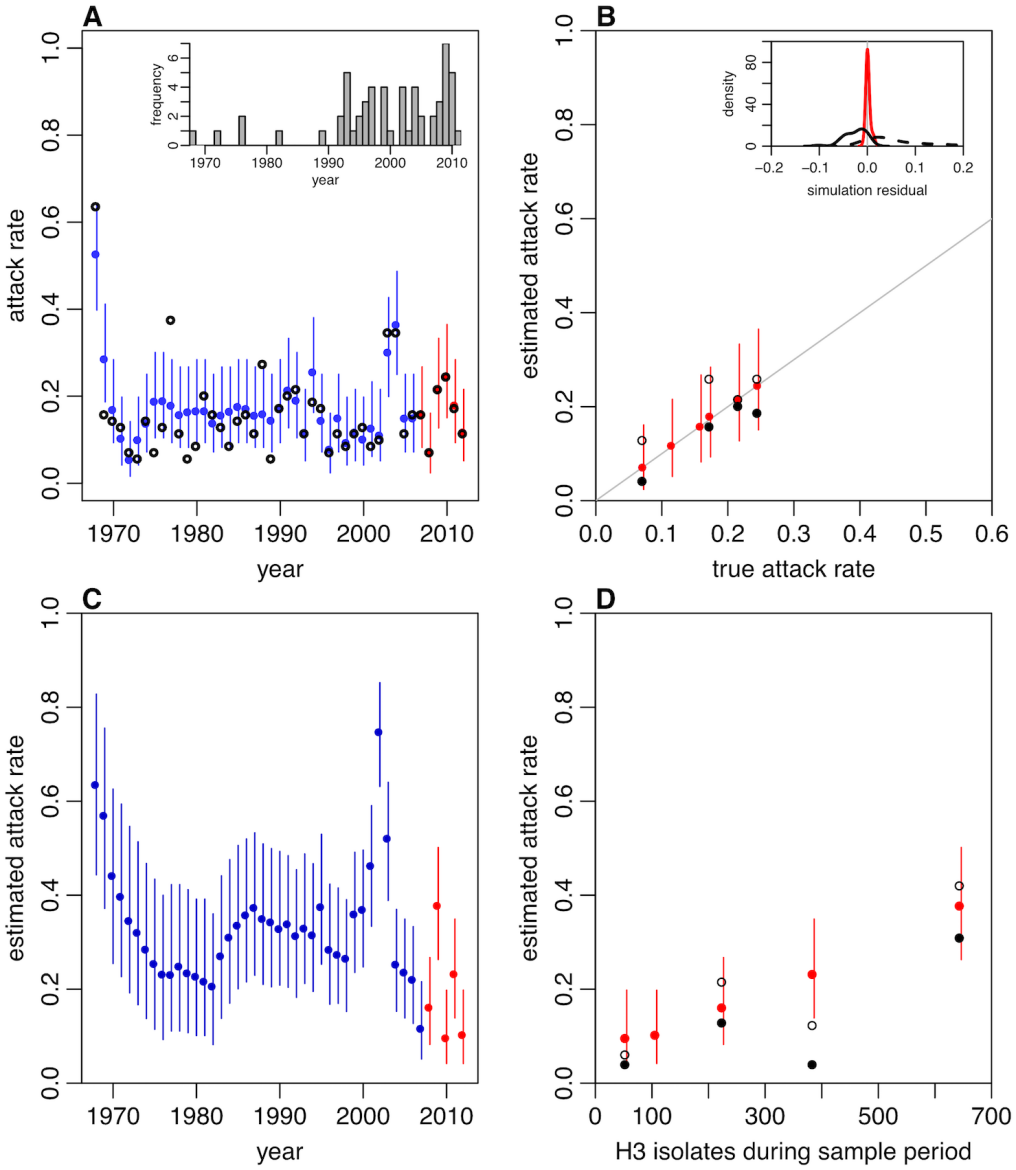
Estimation of influenza A/H3N2 attack rates. (A) Inference of attack rates using simulated data for 69 participants, with same strains as tested in the Ha Nam data. Main plot: Blue lines show the estimated attack rate with binomial confidence interval; red lines show the attack rates in years when samples were taken; black circles show the true attack rate in the original simulation. Inset: year of circulation for the 57 test strains used, which included repeats in some years. (B) Main: accuracy of attack rate estimates in (A) was high for years in which serological samples were collected (shown as red dots). Hollow black points show the attack rate based on a 2-fold rise in titre against strain in that year (points shown for years 2008–2011, which had sufficient test strains or samples to perform this calculation); solid points show the attack rate based on a 4-fold rise. Inset: distribution of differences between estimated and actual attack rates in same years across 12 simulation studies. Red line indicates estimates from model; dashed black line shows estimates based on a 2-fold rise in titre; solid black line shows estimates based on a 4-fold rise. (C) Proportion of the Vietnam study population estimated to have been infected in each year, based on real data. Blue lines show the estimated attack rate with binomial confidence interval; red lines show attack rates in years when samples were taken. (D) Accuracy of attack rate estimates using different methods. Plot shows model estimates of attack rates in 2008–2012 (red points in (C)) and number of positive H3 isolates reported in Vietnam during the same intervals as the samples were taken. Hollow black points show the attack rate based on a 2-fold rise in titre against strain in that year (point not shown for 2012, as no test strains for this year were available, so a rise could not be calculated); solid points show the attack rate based on a 4-fold rise.

As well as examining antibody dynamics, we reconstructed historical annual attack rates. In simulation studies, the model could accurately recover attack rates from Vietnam-like serological data, particularly for recent years (Fig 3A). The reduced accuracy of estimation in earlier years reflected the limited coverage of test strains during this period (Fig 3A, inset). Estimates of attack rates based on the traditional gold standard of a 4-fold rise in titre underestimated the actual simulated values (Fig 3B), and an overestimate was obtained if a 2-fold rise in titre was considered instead [18]. This suggests that commonly used metrics could substantially bias estimates of population-level attack rates and hence conclusions about the potential extent of herd immunity and required vaccination coverage. In contrast, estimates from our joint inference framework consistently recovered the true simulated infection dynamics during the period of sampling (Fig 3B, inset). Applying our inference framework to real data from Vietnam to estimate annual attack rates (Fig 3C), we found that estimates were consistent with observed epidemiological dynamics in Vietnam between 2008 and 2012, as measured by the number of influenza A/H3N2 isolates during the testing period (Fig 3D). The correlation between model estimates and observed values was *ρ* = 0.996 (*p* < 0.001), with a weaker association when a 2-fold rise (*ρ* = 0.862, *p* = 0.14) or 4-fold rise (*ρ* = 0.799, *p* = 0.20) was used to estimate attack rates. The model-estimated attack rate for 2002 was significantly larger than surrounding years (Fig 3C). However, this is consistent with the larger clinical attack rates for H3N2 that coincided with the emergence of A/Fujian/411/2002(H3N2)-like influenza strains [26], and an attack rate of 70% would suggest a reproduction number of around 1.8, using a simple epidemic model [27]. Most of the uncertainty in attack rate estimates resulted from individuals with multiple estimated infections; there was little variation in estimated number of infections when individuals had fewer than around 8 median infections (S12 Fig). Based on the median numbers of estimated infections and years at risk, we estimated a median annual risk of infection of around 20% during the period 1968–2012.

## Discussion

Our analysis shows that detailed mechanistic insights can be gained from longitudinal data by jointly considering individual infection histories and antibody dynamics acting at multiple timescales. Building on previous analyses [13,17,28,29], we estimated that nonprimary influenza exposures generate a large, short-lived broad humoural response and a smaller persistent narrow response, with each accumulating and degrading to different degrees over the course of a human lifetime. As well as quantifying processes that shape the antibody response against different influenza strains, our results suggest that accounting for such dynamics leads to improved estimation of population attack rates.

The short-lived broad response, which we estimated makes the largest contribution to titres following infection, is likely to influence selection pressure imposed on the virus as a result of population immunity; it has been suggested that such short-term nonspecific immunity could explain the constrained genetic diversity of circulating influenza viruses [4]. Measuring this ‘dynamic herd immunity’ would have implications for use of serology to investigate the evolutionary dynamics of influenza and hence identify potential vaccine strain candidates [28, 30, 31]. Because we could infer broadly cross-reactive memory responses from HI titres, such responses likely target the influenza HA head domain [32] rather than conserved epitopes in the stalk [7, 33]. This is consistent with studies that have identified such conserved epitopes in the HA head [34, 35]. If a large proportion of a population had recently experienced infection, they may exhibit a short-term antibody response against such epitopes. If these responses provided protective immunity, it would reduce the transmission potential of strains occupying a large region of antigenic space. However, these strains may become more transmissible as the short-term response wanes to leave a more specific long-term response.

Additional insight into the temporal antibody dynamics described in Fig 2 could be generated directly using modern methods of sorting and sequencing individual B cells [36]. During nonprimary infections, existing memory B cells generated during prior infections, which are genetically diverged from germline B cells, are rapidly stimulated (S13 Fig). These B cells may reach high peripheral frequencies rapidly but, on average, have lower avidity against the current strain than they would have had against that host’s previous infections [37]. If a serum sample were tested at this point, it may therefore exhibit a large, broadly cross-reactive response similar to that observed in Fig 2E. However, there will be competing demands on these cell lines to produce antibodies and possibly to differentiate further to increase their avidity. In addition to these memory cells, there is also the potential for the stimulation of germline B cells, which may take longer to achieve functional peripheral frequencies but have higher avidity [38]. When observed early in the infection, these new lineages would be much more similar to germline B cells and would form fewer phylogenetic clades per sorted cell than the rapid response. Later during infection, cells making up the persistent response would be at higher frequencies and be more differentiated but still form only few clades. Antigenic seniority [13] may arise because novel lineages during later life infections have to compete with existing lineages for antigenic stimulation [39, 40]. After infection, the memory frequency of the B cells making up the broad response likely returns to preinfection levels, and the new B cells establish new subordinate memory populations that are less broadly cross-reactive. The aggregate effect of these mechanisms that would be observed in serological samples is consistent conceptually with the results we have presented (S13 Fig).

There are some limitations to our analysis. First, we assumed that the antigenic evolution of influenza results in a sequence of strains that follow a smooth path in a two-dimensional antigenic space (S1 Fig). However, it has been suggested that the multiple epitopes of the influenza HA mean that the antigenic relationship between strains may not be necessarily explained by a gradual accumulation of antigenic distance over time [34, 35, 41]. As we are analysing serological samples taken from individuals without a fully known exposure history, it would be challenging to infer antigenic relationships between historical strains without any a priori assumptions about antigenic space [28]. If we were to try and estimate the antigenic locations of circulating A/H3N2 strains from 1968 to 2008 in a two-dimensional space—rather than assume their locations as we did—it would add 40 × 2 = 80 parameters to the model, which would not be identifiable from the data we used in this study. If we imposed no constrains on the dimension of antigenic space, we would have to estimate up to 40 × 40 = 1,600 pairwise distances between strains. One aim for future research would be to design a cohort study with sufficient information on prior influenza exposures to infer antigenic distances between specific historical strains; the model presented here could then be used to compare the relative explanatory power of different assumptions about the path of antigenic evolution.

Second, we analysed data from 2 types of assay. HI and microneutralisation tests capture 2 different aspects of the antibody response—namely, anti-haemagglutination activity and neutralising effects—and hence, measured titres are not directly comparable [6]. Although there was a high correlation between HI and microneutralisation titres observed in the China data we analysed [22], we fitted models independently to each dataset, meaning that any differences in assay characteristics would be reflected as differences in our parameter estimates (Table 2). With a larger number of repeat serological samples tested using both assays, it would be possible to compare specific aspects of the assay dynamics in more detail.

The modelling approaches we have described could also be employed in evaluating the effectiveness of influenza vaccination strategies, which depends on an ability to reliably infer population attack rates. For example, metrics that systematically overestimate influenza attack rates could result in underpowered studies. Moreover, the broader concept of multiple timescales of antibody response would have potential implications for the design of innovative vaccines, such as highly valent vaccines [10]. If broad responses have shorter durations than narrow responses, then the trade-off between current vaccines and other proposed candidates may be time dependent. Participants in trials of novel influenza vaccines should therefore be followed up over multiple seasons so that the dynamics of their immune response to both vaccination and natural infection can be assessed. At best, such vaccination against influenza A/H3N2 may stimulate a similar response to natural infection. However, there is evidence that vaccine-mediated immunity wanes quickly [42], that vaccine effectiveness declines after multiple immunisations [43], and that broad response against a novel subtype fades after repeated vaccination [44]. With appropriate data on serology and vaccination history, the differences in dynamics between the two processes could be elucidated using the model structure we have presented.

As well as examining differences in vaccination-mediated immunity and antibody response following natural infection, future empirical studies could refine our estimate of the short-term response by collecting serological samples at intervals of less than 1 year. Alternatively, or additionally, having information on timing of confirmed influenza infection between sample collections would make it possible to constrain possible infection events and hence improve estimates of short-term dynamics. In our model, we also accounted for individual-level heterogeneity in titres by including normally distributed error in our observation model. Our results suggest that this error parameter is well identified (S1 Table), but it would be challenging to examine other potential heterogeneity in antibody responses—such as age-specific biases—in more detail with the data available without making strong assumptions about the nature of such heterogeneity.

Our inference approach could be used in the future to guide the design of studies to infer key aspects of antibody dynamics or to estimate historical attack rates. Joint analysis of infection history and antibody dynamics could provide more accurate information about infection rates, particularly in the years preceding sample collection, and inform studies that rely on robust attack rate estimates. As a result, such methods could help ensure that serological studies to examine influenza immunity profiles have adequate statistical power to test hypotheses and identify key mechanistic processes. Our approach is also likely to be applicable to other cross-reactive pathogens, such as dengue fever and Zika viruses [45].

## Materials and methods

### Serological data

We used 2 publicly available datasets in our analysis. In the southern China data, cross-sectional serology was taken in 2009 from 151 participants in the Guangdong province in southern China and tested using HI and microneutralisation assays against a panel of 9 strains: 6 vaccine strains (A/Hong Kong/1/1968, A/Victoria/3/1975, A/Bangkok/1/1979, A/ Beijing/353/1989, A/Wuhan/359/1995, and A/Fujian/411/2002) and 3 strains that circulated in southern China in recent years preceding the study (A/Shantou/90/2003, A/Shantou/806/2005, and A/Shantou/904/2008) [13, 21]. The Vietnam data included longitudinal serology collected between 2007 and 2012 from 69 participants in Ha Nam [23], with sera tested using HI assays against a panel of up to 57 A/H3N2 strains isolated between 1968 and 2008 [10]. All of the Vietnam participants were unvaccinated against influenza, and 19% of the southern China participants reported prior influenza vaccination. In analysis of both datasets, we represented antibody responses by log titre. For a titre dilution of 10 ≤ *D* ≤ 1,280, log titre was defined as ${\text{log}}_{2}\left(\frac{D}{10}\right)+1$. The minimum detectable titre in both datasets was 10, so a dilution <10 was defined to have a log titre of 0. The maximum observable titre in both datasets was 1,280, which corresponded to a log titre of 8. There were 9 possible observable log titres in our analysis, ranging from 0 to 8. The antigenic summary path used to represent strains in our analysis was generated by fitting a two-dimensional smoothing spline through 81 points representing the published estimated locations of strains in ‘antigenic space’ [10] (S1 Fig). The positions of strains in such a space depends on the distance between influenza antigens and reference antisera as measured by titre in an HI assay [20]. In the model, we assumed that strains circulating between 1968 and 2012 were uniformly distributed along this summary path.

### Model of expected titre given infection history

We expanded and refined a previous modelling framework designed for cross-sectional data [17] to include short- and long-term dynamics. For an individual who had previously been infected with strains in the set *X*, the expected log titre against strain *j* depended on 5 specific antibody processes:

1. Long-term boosting from infection with homologous strain. If an individual had been infected with only one strain, they would exhibit a fixed log titre against that strain, controlled by a single parameter, *μ*_1_.
2. Antigenic seniority acting via suppression of subsequent responses as a result of prior immunity. The titre against a particular strain was scaled by a factor *s*(*X*, *m*) = max{0,1 − *τ*(*N*_*m*_ − 1)}, where *N*_*m*_ is the number of the strain in the infection history (i.e., the first strain is 1, the second is 2, etc.), |*X*| is the total number of infections, and *τ* was a parameter to be fitted.
3. Cross-reactivity from antigenically similar strains. As titres were on a log scale, we assumed the level of cross-reaction between a test strain *j* and infecting strain *m* ∈ *X* decreased linearly with antigenic distance. This was controlled by *d*_1_(*j*, *m*) = max{0,1 − *σ*_1_*δ*_*mj*_}, where *δ*_*mj*_ was the two-dimensional euclidean antigenic distance between strains *j* and *m* (S1 Fig), and *σ*_1_ was a parameter to be fitted.
4. Short-term boosting, which waned over time. For an infecting strain *m*, this process was controlled by *μ*_2_*w*(*m*) = *μ*_2_max{0,1 − *ωt*_*m*_}, where *μ*_2_ was a boosting parameter, *ω* was a waning parameter to be fitted, and *t*_*m*_ was the number of years since infection with strain *m*. We constrained *ω* ≤ 1 when fitting the model to ensure identifiability, as *ω* = 1 or *ω* > 1 implies that *w*(*m*) = 0 for all *t*_*m*_ > 0.
5. Cross-reactivity for the short-term response. The level of cross-reaction between a test strain *j* and infecting strain *m* was given by *d*_2_(*j*, *m*) = max{0,1 − *σ*_2_*δ*_*mj*_}, where *δ*_*mj*_ was the antigenic distance between strains *j* and *m*, and *σ*_2_ was a parameter to be fitted.

To combine the 5 processes in the model, we assumed that the expected log titre individual *i* had against a strain *j* was a linear combination of the responses from each prior infection:

$$\lambda_{ij}=\underset{m\in X}{\sum }s(X,m)\left[\mu_{1}d_{1}(j,m)+\mu_{2}w(m)d_{2}(j,m)\right].$$ (1)

Depending on parameter values, our model could incorporate several specific mechanistic features, including long-term response only (*μ*_2_ = 0), waning response only (*μ*_1_ = 0), or long-term/short-term boosting independent of a cross-reactive memory response (*σ*_1_, *σ*_2_ = 0).

### Observation model and likelihood function

For an individual *i* who was infected with strains in the set *X*, we assumed their true titre against strain *j* titre followed a normal distribution with mean *λ*_*ij*_, standard deviation *ε*, and cumulative distribution function *f*(*x*). The observed distribution of titres was censored to account for integer-valued cutoffs. The likelihood of observing titre *k* ∈ {0,…,8} given history *X* and parameter set *θ* was therefore as follows:

$$L(k|\theta ,X)=\{\begin{array}{ll} f(x<1) & \text{if}k=0;\\ f(k\leq x<k+1) & \text{if}1\leq k<8.\\ f(x\geq 8) & \text{if}k\geq 8; \end{array}$$ (2)

Note that there are several key differences between the model framework presented here and the one we described previously [17]. These changes were designed to increase model flexibility and biological detail: infections occur annually, rather than within antigenic epochs; we assume waning and cross-reaction decays linearly with log titre, rather than exponentially; the observation model explicitly accounts for censoring, rather than using a discrete observation distribution; and strains are explicitly located in an antigenic space, rather than distance represented temporally. It is therefore not possible to directly compare parameter estimates from this model with the previous framework, because the values need to be interpreted in the context of the specific assumptions in the underlying models.

### Parameter estimation and model comparison

We fitted the model independently to each serological dataset using Markov chain Monte Carlo (MCMC). Using the likelihood function in Eq 2, we jointly estimated *θ* across all individuals and estimated *X* for each individual via a Metropolis-Hastings algorithm. We used uniform positive priors for all *θ* parameters, with *ω* constrained to be in the interval [0,1), as it would not be identifiable on timescales of less than a year, given annual sample collection. If individual sera were collected in more than 1 year, parameters were jointly estimated across all test years. We used a data augmentation approach to estimate individual infection histories. Every second iteration, we resampled model parameters, which were shared across all individuals, and performed a single Metropolis-Hastings acceptance step. On the other iterations, we resampled infection histories for a randomly selected 50% of individuals. These histories were independent across individuals, so we performed a Metropolis-Hastings acceptance step for each individual separately.

To ensure the Markov chain was irreducible, resampling at each step involved one of the following: addition of infection in some year, removal of infection in some year, or moving an infection from some year to another [46]. We also used adaptive MCMC to improve the efficiency of mixing: at each iteration, we adjusted the magnitude of the covariance matrix used to resample *θ* to obtain an acceptance rate of 0.234 [47]. As we had data on participants’ individual ages in the southern China data, we constrained potential infections in the model to years in which participants would have been alive. To estimate the median and 95% credible interval for attack rates, we sampled from the posterior distribution of infection histories and calculated the total participants who were estimated to have been infected in each year at each iteration. The resulting attack rates were therefore implicitly binomially distributed. The model was implemented in R version 3.3.1 and C and used the Rcpp and doMC packages. Source code and data are available at https://github.com/adamkucharski/flu-model/.

Correlation plots indicated that all parameters in the full model were identifiable (S14 Fig), with ESS above 200 (S1 Table). We included both short- and long-term dynamics when fitting to longitudinal data, because the model that included short-term antibody dynamics performed substantially better than the model with long-term dynamics only. As the model with long-term-only dynamics was a nested version of the model with both short-term and long-term dynamics (i.e., with *μ*_2_ = 0), we used the Savage-Dickey density ratio (SDDR) to approximate the Bayes factor [48]. The prior for *μ*_2_ was flat, and the posterior density for *μ*_2_ did not include 0 (S9 Fig), which meant the SDDR was 0. This indicated overwhelming support for the more complex model. The more complex model also required a lower variance in the observation model distribution to fit the data: the estimated error term, *ε*, was 1.29 (95% CrI: 1.27–1.31) when the model with short-term dynamics was fitted to longitudinal data and 1.39 (1.37–1.41) when short-term dynamics were omitted (S9 and S15 Figs). We also compared model performance using a training/test approach. We fitted the models with and without short-term dynamics to the same training dataset, constructed by randomly selecting 90% of the titre results available for each participant in the Vietnam dataset. We then used these fitted models to predict titres in a test dataset, which consisted of the remaining 10% of titres. The mean absolute error between observed and median predicted titres in the test dataset was 1.18 for the model with short-term response and 1.23 for the model without; the root-mean-square error (RMSE) was 1.54 for the short-term response model and 1.61 for the simpler model. However, it is worth noting that inclusion of a short-term response would not have been expected to improve predictions for all participants and strains: short-term dynamics only influence titres for participants who have evidence of infection shortly prior to sample collection, and titres against recently isolated strains will be more influenced by the short-term response than titres against older strains.

### Simulation study

In our simulation study, we first generated simulated influenza attack rates between 1969 and 2012 using a lognormal distribution with mean 0.15 and standard deviation 0.5. For 1968, we used a lognormal distribution with mean 0.5 to reflect higher incidence in the pandemic year [49]. Using these simulated attack rates, we generated individual infection histories for 69 participants using a binomial distribution and then generated observed individual-level titres against the same strains as in the Vietnam dataset using our titre model. Simulated samples were tested each year between 2007 and 2012. Based on the parameters estimated using real data, we assumed *μ*_1_ = *μ*_2_ = 2, *τ* = 0.05, *ω* = 0.75, *σ*_1_ = 0.2, *σ*_2_ = 0.1, and *ε* = 1 in simulations. Finally, we used the model to reestimate infection history and attack rates. For Fig 3B inset, we simulated 12 independent sets of observed titres and then inferred the proportion of the population infected in the 4 years between 2008 and 2011 inclusive. The resulting distribution of model residuals (i.e., estimated minus actual simulated value) for these 48 data points was plotted as kernel density plots.

### Epidemiological data

Reported influenza A/H3N2 activity in Vietnam was obtained from the WHO FluNet database [50] (S16 Fig). We aggregated reports into temporal windows based on dates of serological sample collection [10] and used the cumulative number of isolates in each period to compare observed activity with model estimates. To calculate attack rates from the model outputs, we scaled the posterior distribution of total number of infections across all participants for each year between 1968 and 2012 by the proportion of participants who were alive in that year, which we calculated based on the age distribution of participants. This produced the estimates in Fig 3C and 3D.

## Supporting information

S1 Fig Schematic of model. (A) Following infection, individuals have a short- and long-term boost in log titre against a homologous strain. (B) The short-term response wanes to leave a long-term persistent boost in log titre. (C) Following infection, individuals also have a short- and long-term boost in log titre against strains that are nearby in antigenic space (S2 Fig). The breadth of cross-reaction may be different for the short- and long-term response. (D) Subsequent infections may generate lower levels of boosting than generated against strains encountered earlier in life, as a result of antigenic seniority. (E) Annotated version of model, as specified in Eq 1 of the Materials and methods.
(TIFF)

S2 Fig Assumed antigenic locations of historical strains in model between 1968 and 2012. These locations were generated by using a spline to estimate a ‘summary path’ of influenza antigenic drift (blue line) from the antigenic locations of strains isolated during this period (shown as grey dots), following from previous work [10]. Assumed locations shown as blue dots.
(TIFF)

S3 Fig Simulation study posterior results. Inference performed using simulated data for 69 participants, with same strains as tested in the Ha Nam data (57 in total, including repeats in some years). (A) Convergence plots for 4 MCMC runs are shown. Note that each run used a different simulation dataset, so the likelihoods are not directly comparable. (B) Comparison of simulated and true attack rates for one of the chains. Blue lines show estimated attack rate with binomial confidence interval; red lines show attack rates in years when samples were taken. Similar results were obtained for all 4 chains. (C) The accuracy of attack rate estimates was better for recent years (shown as red dots), which were more densely sampled in the serological data. MCMC, Markov chain Monte Carlo.
(TIFF)

S4 Fig Selection of 15 individual estimated histories for simulated data. Red points show observed titre. Blue lines show median titre in fitted model, with blue regions showing 50% and 95% MCMC credibility intervals. Black lines show samples from the posterior distribution of individual infection histories, with opacity indicating the probability of infection (i.e., proportion of MCMC samples that estimated infection in that year). Green lines show true years of infection in simulation. Final column shows distribution of total estimated infections |*X*|, with simulated value shown by green line. MCMC, Markov chain Monte Carlo.
(TIFF)

S5 Fig Selection of 50 individual estimated histories for A/H3N2 FluScape HI data. For each participant, distribution of total estimated infections |*X*| is also shown. HI, haemagglutination inhibition.
(TIFF)

S6 Fig Selection of 25 individual estimated histories for A/H3N2 Vietnam HI data. Final column shows distribution of total estimated infections |*X*|. HI, haemagglutination inhibition.
(TIFF)

S7 Fig MCMC diagnostics for 4 chains fitted to A/H3N2 FluScape microneutralisation data. Dashed line shows burn-in period. MCMC, Markov chain Monte Carlo.
(TIFF)

S8 Fig MCMC diagnostics for 4 chains fitted to A/H3N2 FluScape HI data. Dashed line shows burn-in period. HI, haemagglutination inhibition; MCMC, Markov chain Monte Carlo.
(TIFF)

S9 Fig MCMC diagnostics for 4 chains fitted to A/H3N2 Vietnam HI data. Dashed line shows burn-in period. HI, haemagglutination inhibition; MCMC, Markov chain Monte Carlo.
(TIFF)

S10 Fig Distribution of estimated number of infections across all individuals using different assays performed on the A/H3N2 China serology. Blue bars, HI data; red bars, microneutralisation data. HI, haemagglutination inhibition.
(TIFF)

S11 Fig Schematic of antibody response following infection. (A) Predicted log titre against a homologous virus following infection, based on 10,000 bootstrap samples from the fitted model, including observation error. Solid line, median; dark shaded region, 50% CrI; light shaded region, 95% CrI. (B) Predicted log titre against a strain located a distance of 5 antigenic units from the infecting virus. (C) Predicted log titre against a strain located a distance of 10 antigenic units from the infecting virus. CrI, credibility interval.
(TIFF)

S12 Fig Distribution of infections and age in the A/H3N2 Vietnam HI dataset. (A) Distribution of estimated number of infections for each participant, with median and 95% credible interval shown. (B) Cumulative distribution of estimated infections for study participants, with median and 95% CrI shown. Values calculated by sampling from the posterior cumulative distribution of total infections for participants. (C) Cumulative distribution of number of years at risk for A/H3N2 infection for study participants (i.e., number of years alive in the period post 1968). HI, haemagglutination inhibition.
(TIFF)

S13 Fig Schematic of 2-armed immune response against sequential influenza viruses. (A) In this simple illustration, each virus has 3 epitopes that can be targeted by monoclonal antibodies. The first infection, with virus A, stimulates distinct populations of memory B cells within the host that produce (B) antibodies with high avidity to epitope 1 and (C–D) antibodies with lower avidity to epitopes 2 and 3. After clearance of virus, these B cell populations decline to an equilibrium level. A serological sample tested at this point (Test 1) would exhibit a long-term response specific to virus A only. Upon secondary infection with virus B—which has epitopes 2 and 3 but with a new epitope 4 in place of epitope 1—the lower-avidity B cell populations are activated, along with (E) a newly stimulated B cell population that has high avidity to epitope 4. However, the virus population is neutralised before these B cells reach the level of earlier B cell populations, which produce the ‘antigenic seniority’ effect. Following the secondary infection, the host would exhibit raised levels of antibodies against epitopes 2 and 3 and hence produce a response even against viruses with only one of these epitopes. This results in a short-lived, broadly cross-reactive serological response (Test 2), which wanes to leave a narrower long-term response (Test 3).
(TIFF)

S14 Fig Correlation plots for parameter estimates using A/H3N2 Vietnam HI data. Pairwise plots show 1,000 MCMC samples from the full joint posterior distribution. HI, haemagglutination inhibition; MCMC, Markov chain Monte Carlo.
(TIFF)

S15 Fig MCMC diagnostics for 4 chains fitted to A/H3N2 Vietnam HI data, using a model without short-term dynamics. Dashed line shows burn-in period. HI, haemagglutination inhibition; MCMC, Markov chain Monte Carlo.
(TIFF)

S16 Fig Vietnam weekly influenza isolates. (A) All influenza isolates reported [50]. (B) A/H3N2 isolates. Red lines show times of serological sampling. (C) Cumulative isolates in each period.
(TIFF)

S1 Table Parameter estimates for models fitted to data from southern China and Vietnam. Median estimate shown, with 95% credible interval in parentheses. ESS for each parameter is also shown, to indicate the extent of autocorrelation in MCMC sampling. ESS, effective sample size; MCMC, Markov chain Monte Carlo.
(PDF)

## Abbreviations

CrI: credibility interval
HA: haemagglutinin
HI: haemagglutination inhibition
MCMC: Markov chain Monte Carlo
RMSE: root-mean-square error
SDDR: Savage-Dickey density ratio
