# Supplementary figures and images for: Timescales of influenza A/H3N2 antibody dynamics
Source: PLoS Biol. 2018 Aug 20;16(8):e2004974. doi: 10.1371/journal.pbio.2004974 (PMC6117086; doi:10.1371/journal.pbio.2004974)

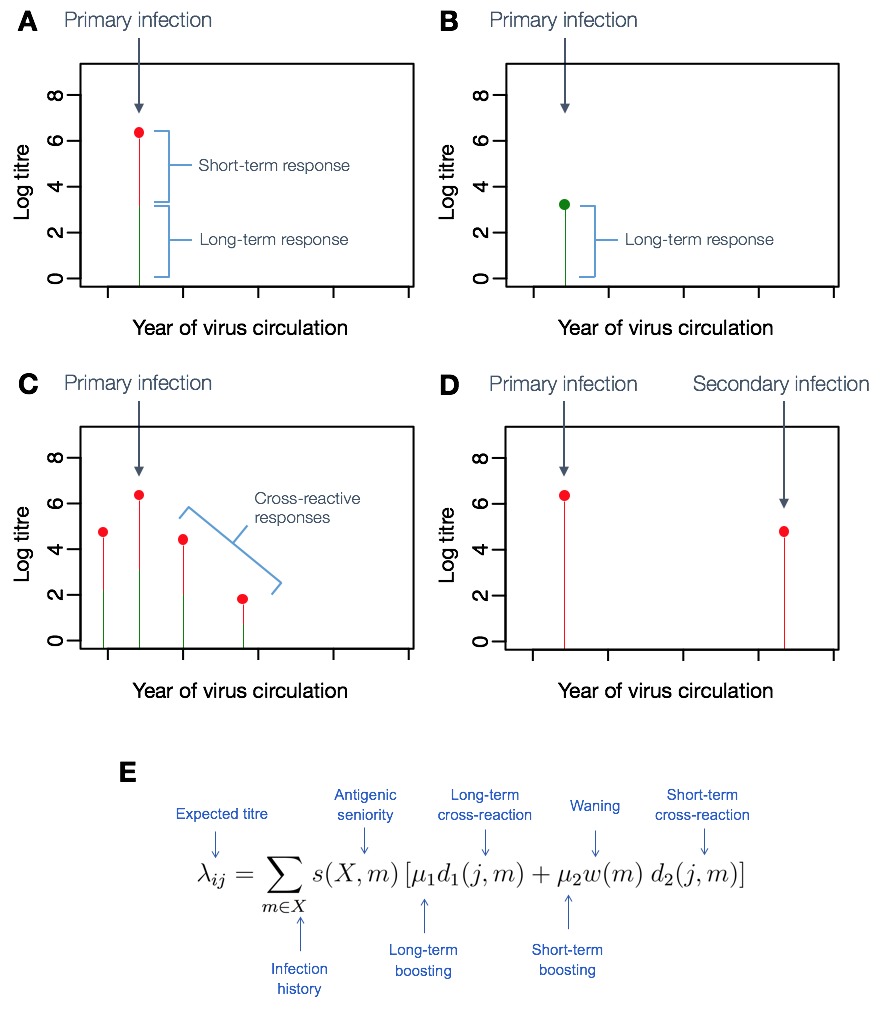

Supplement: S1 Fig — (A) Following infection, individuals have a short- and long-term boost in log titre against a homologous strain. (B) The short-term response wanes to leave a long-term persistent boost in log titre. (C) Following infection, individuals also have a short- and long-term boost in log titre against strains that are nearby in antigenic space (S2 Fig). The breadth of cross-reaction may be different for the short- and long-term response. (D) Subsequent infections may generate lower levels of boosting than generated against strains encountered earlier in life, as a result of antigenic seniority. (E) Annotated version of model, as specified in Eq 1 of the Materials and methods. (TIFF) [file pbio.2004974.s001.tiff]

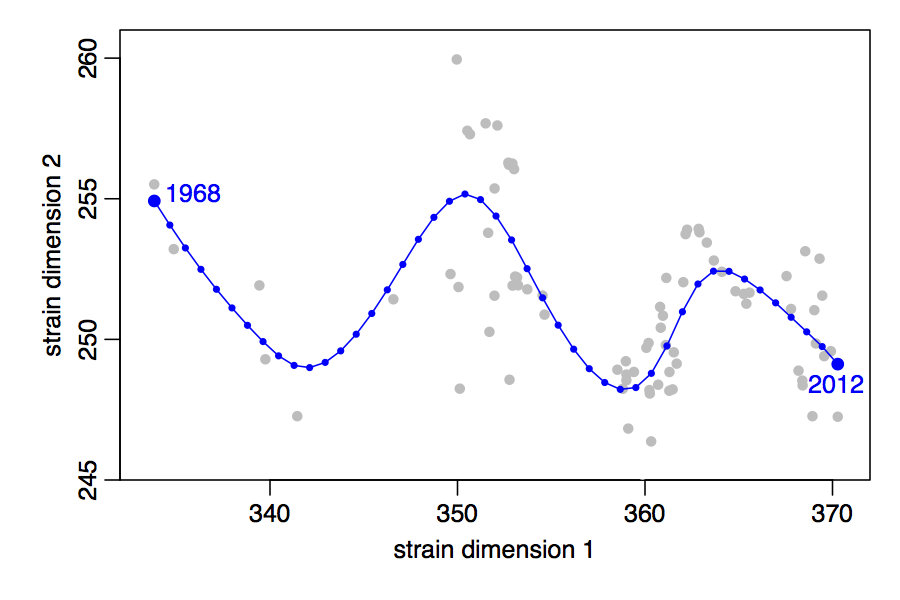

Supplement: S2 Fig — These locations were generated by using a spline to estimate a ‘summary path’ of influenza antigenic drift (blue line) from the antigenic locations of strains isolated during this period (shown as grey dots), following from previous work [10]. Assumed locations shown as blue dots. (TIFF) [file pbio.2004974.s002.tiff]

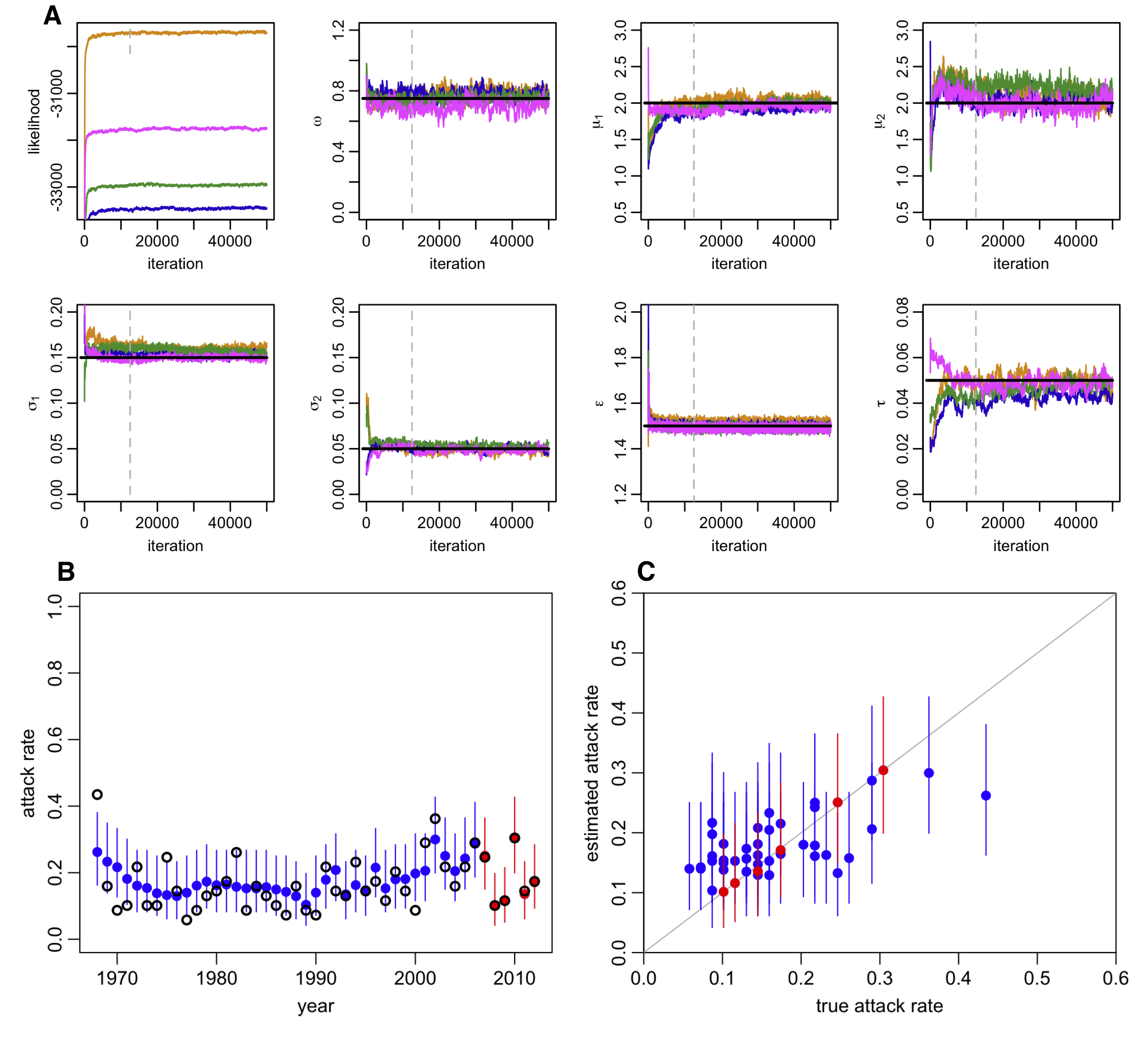

Supplement: S3 Fig — Inference performed using simulated data for 69 participants, with same strains as tested in the Ha Nam data (57 in total, including repeats in some years). (A) Convergence plots for 4 MCMC runs are shown. Note that each run used a different simulation dataset, so the likelihoods are not directly comparable. (B) Comparison of simulated and true attack rates for one of the chains. Blue lines show estimated attack rate with binomial confidence interval; red lines show attack rates in years when samples were taken. Similar results were obtained for all 4 chains. (C) The accuracy of attack rate estimates was better for recent years (shown as red dots), which were more densely sampled in the serological data. MCMC, Markov chain Monte Carlo. (TIFF) [file pbio.2004974.s003.tiff]

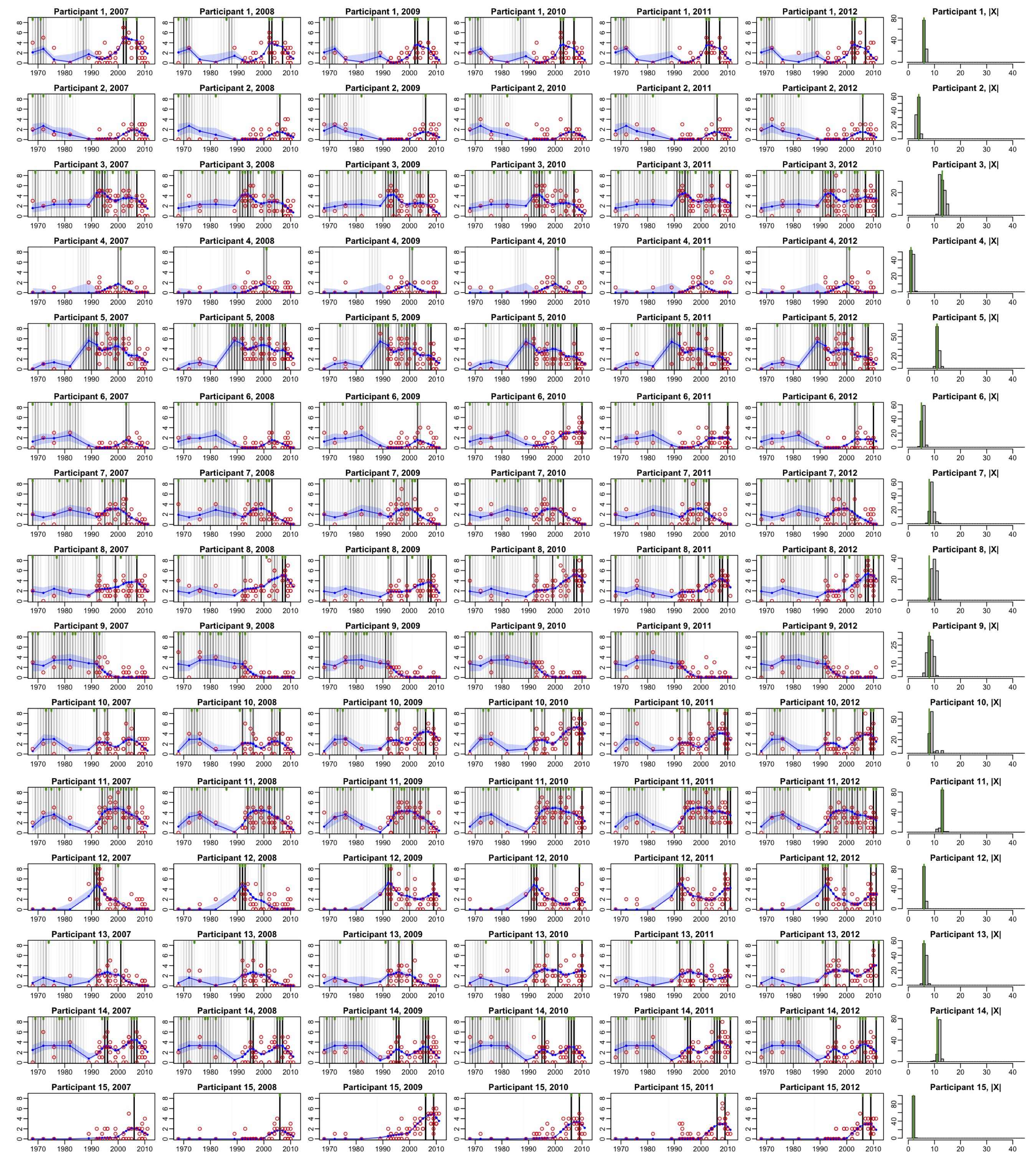

Supplement: S4 Fig — Red points show observed titre. Blue lines show median titre in fitted model, with blue regions showing 50% and 95% MCMC credibility intervals. Black lines show samples from the posterior distribution of individual infection histories, with opacity indicating the probability of infection (i.e., proportion of MCMC samples that estimated infection in that year). Green lines show true years of infection in simulation. Final column shows distribution of total estimated infections |X|, with simulated value shown by green line. MCMC, Markov chain Monte Carlo. (TIFF) [file pbio.2004974.s004.tiff]

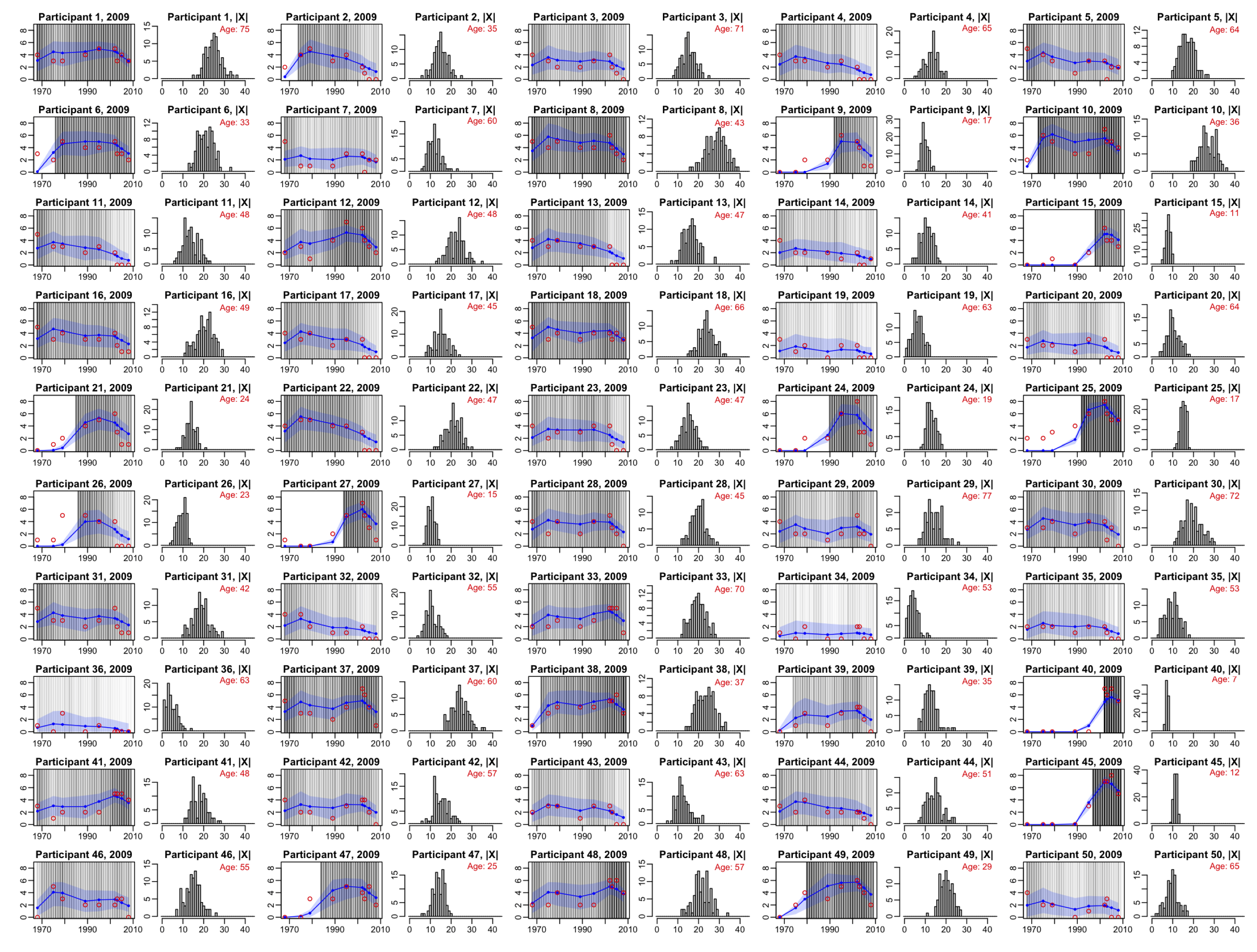

Supplement: S5 Fig — HI, haemagglutination inhibition. (TIFF) [file pbio.2004974.s005.tiff]

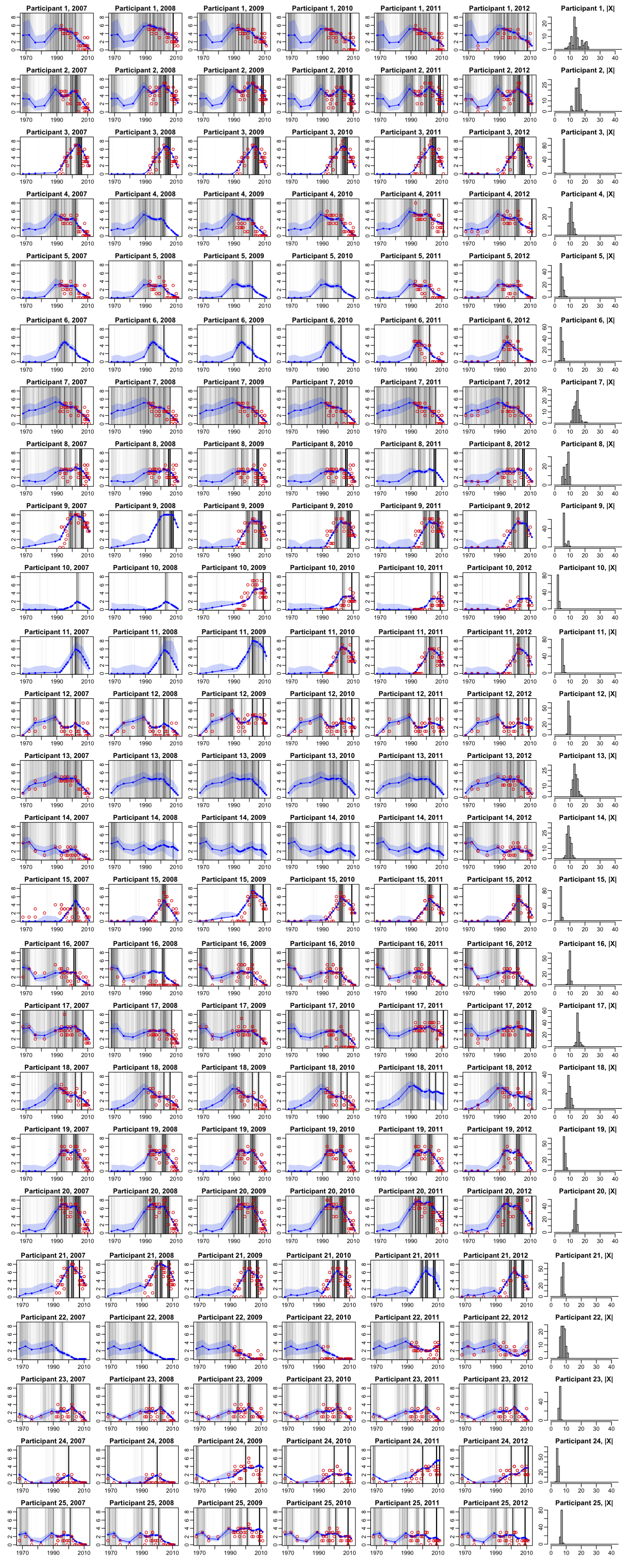

Supplement: S6 Fig — HI, haemagglutination inhibition. (TIFF) [file pbio.2004974.s006.tiff]

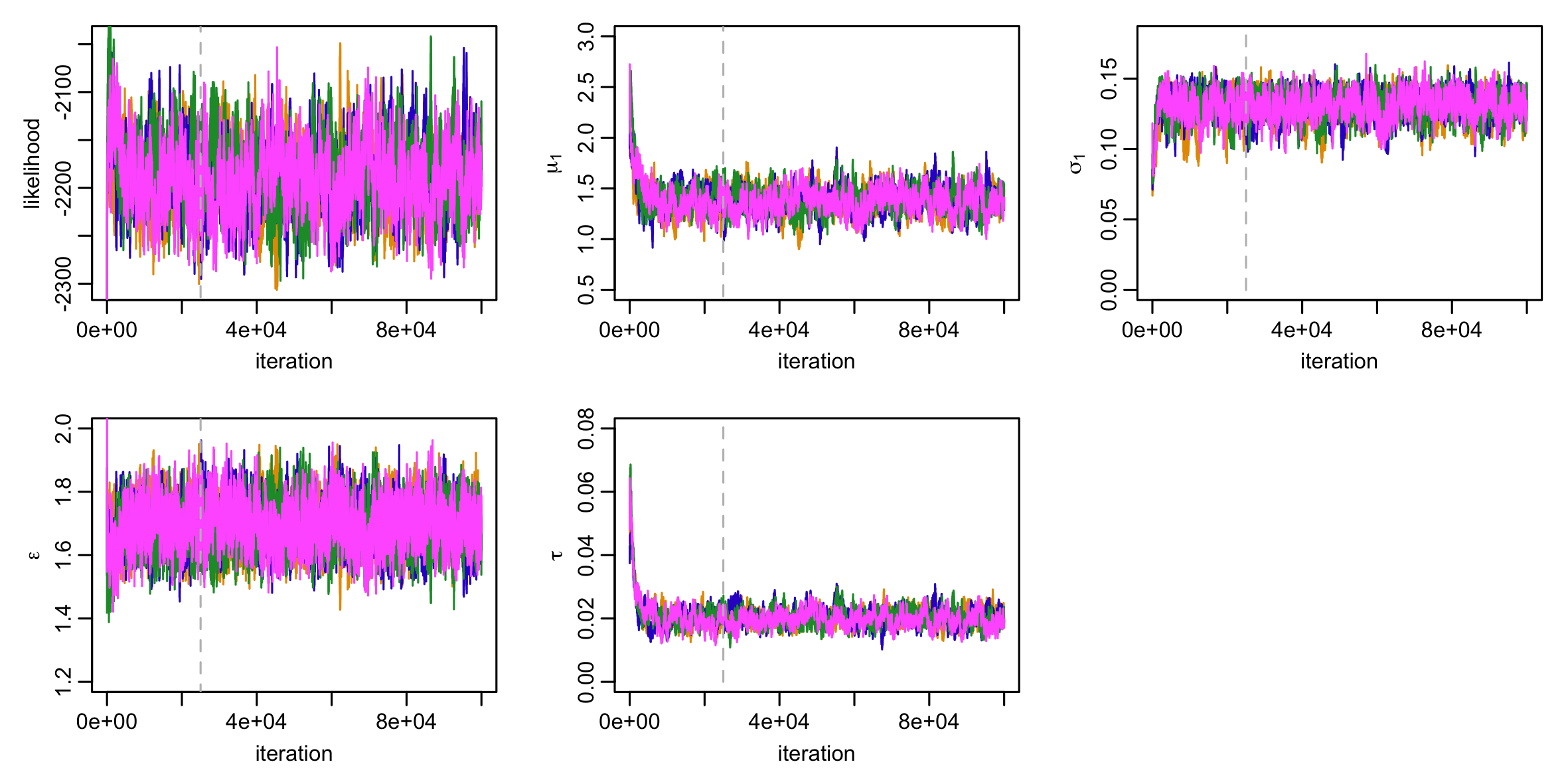

Supplement: S7 Fig — Dashed line shows burn-in period. MCMC, Markov chain Monte Carlo. (TIFF) [file pbio.2004974.s007.tiff]

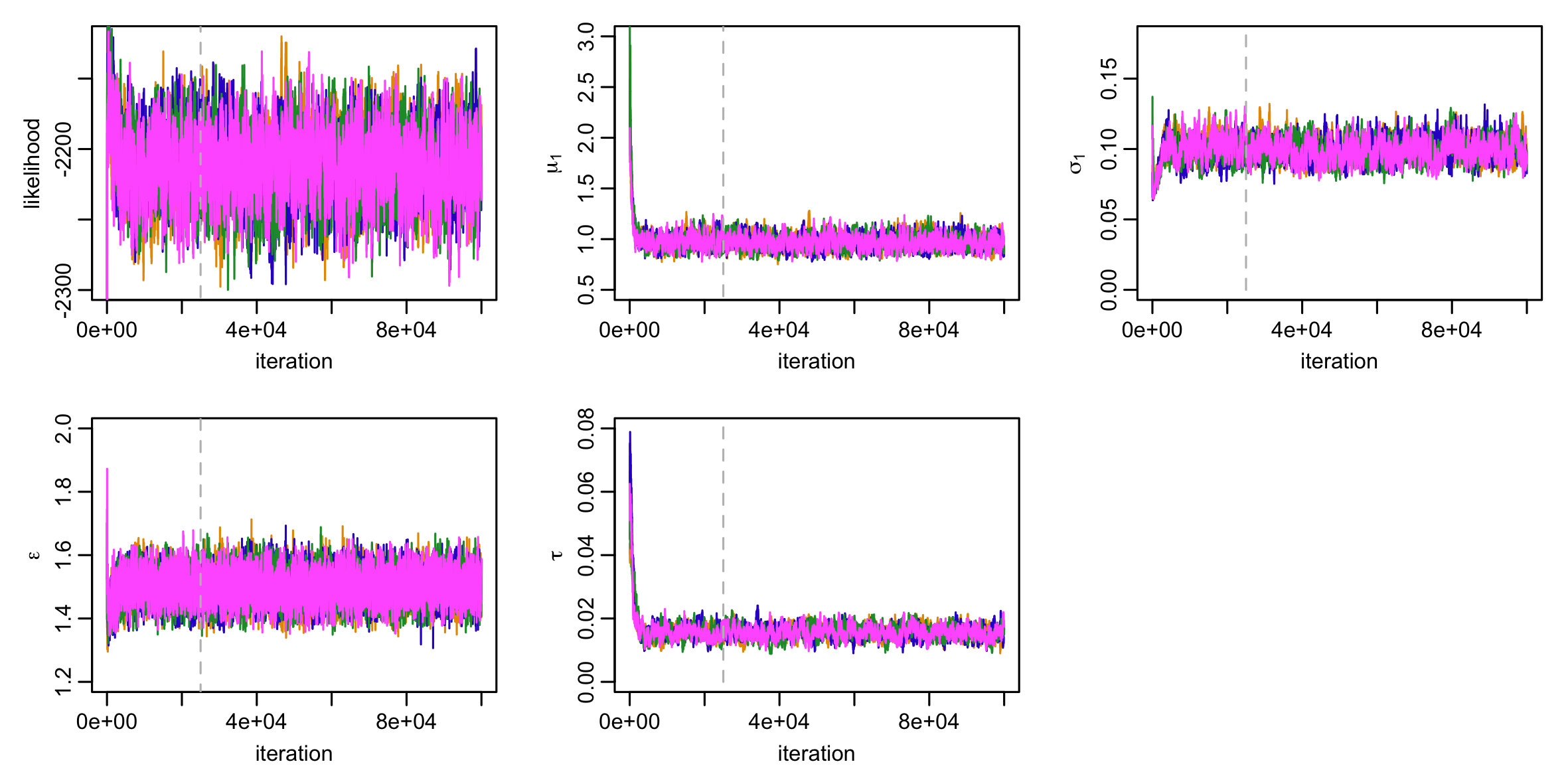

Supplement: S8 Fig — Dashed line shows burn-in period. HI, haemagglutination inhibition; MCMC, Markov chain Monte Carlo. (TIFF) [file pbio.2004974.s008.tiff]

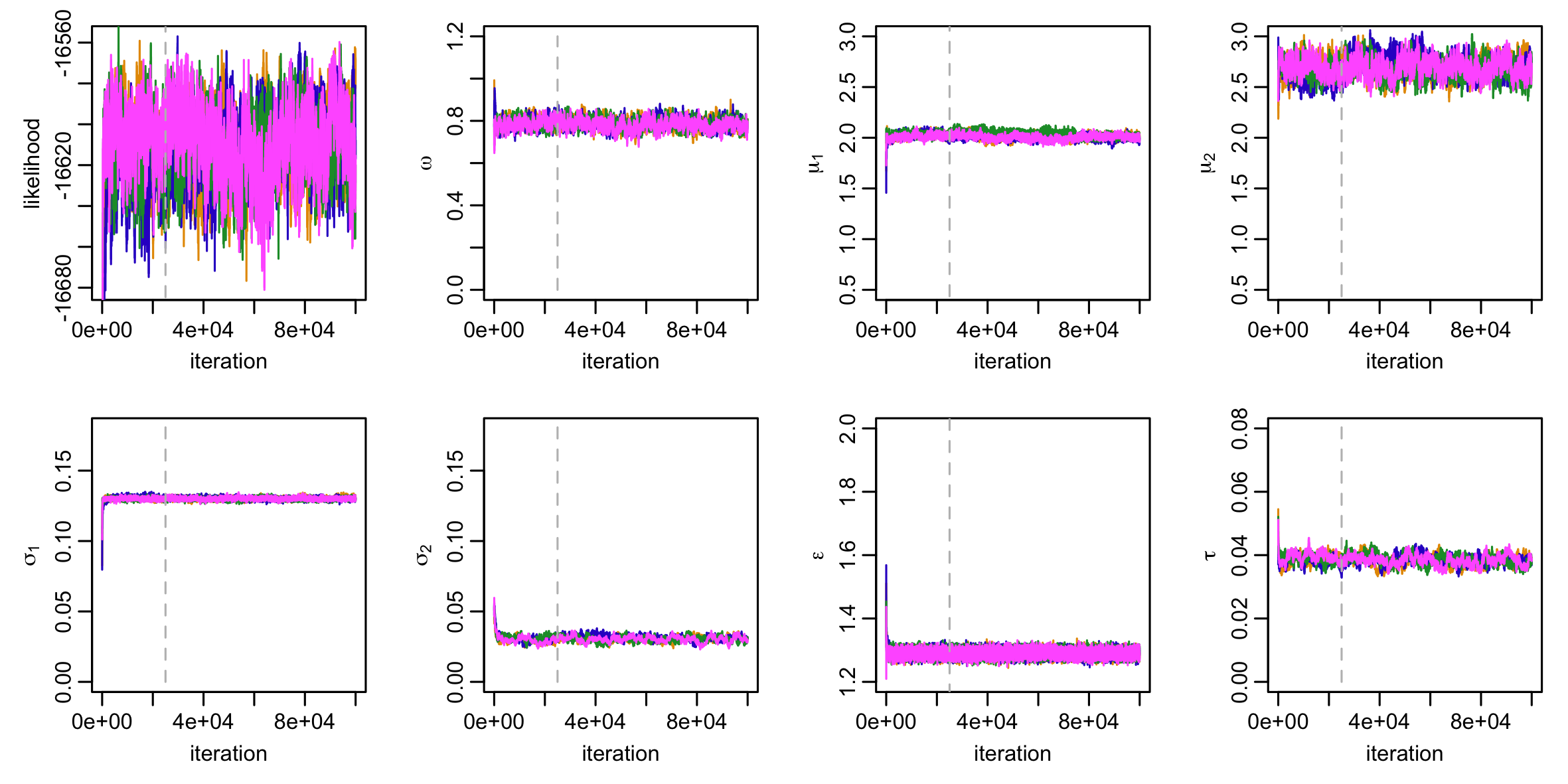

Supplement: S9 Fig — Dashed line shows burn-in period. HI, haemagglutination inhibition; MCMC, Markov chain Monte Carlo. (TIFF) [file pbio.2004974.s009.tiff]

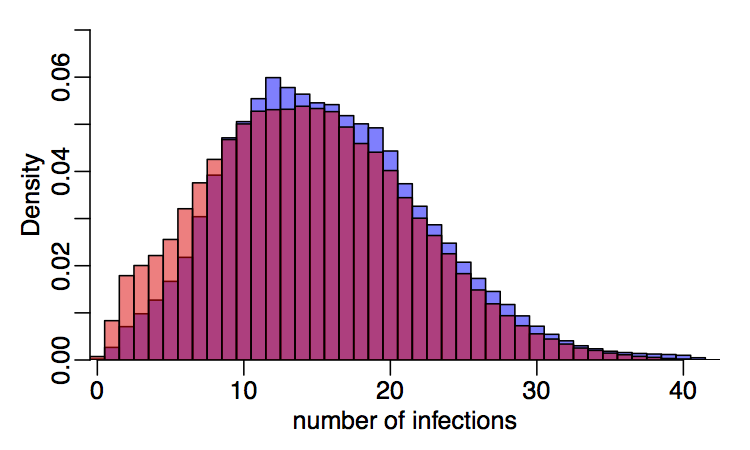

Supplement: S10 Fig — Blue bars, HI data; red bars, microneutralisation data. HI, haemagglutination inhibition. (TIFF) [file pbio.2004974.s010.tiff]

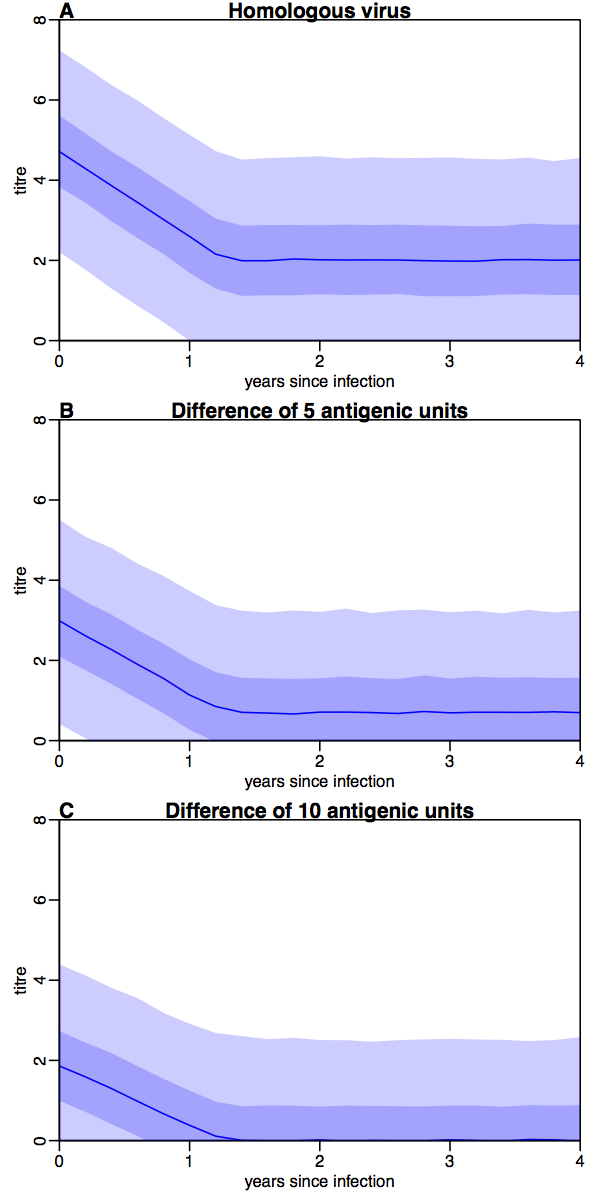

Supplement: S11 Fig — (A) Predicted log titre against a homologous virus following infection, based on 10,000 bootstrap samples from the fitted model, including observation error. Solid line, median; dark shaded region, 50% CrI; light shaded region, 95% CrI. (B) Predicted log titre against a strain located a distance of 5 antigenic units from the infecting virus. (C) Predicted log titre against a strain located a distance of 10 antigenic units from the infecting virus. CrI, credibility interval. (TIFF) [file pbio.2004974.s011.tiff]

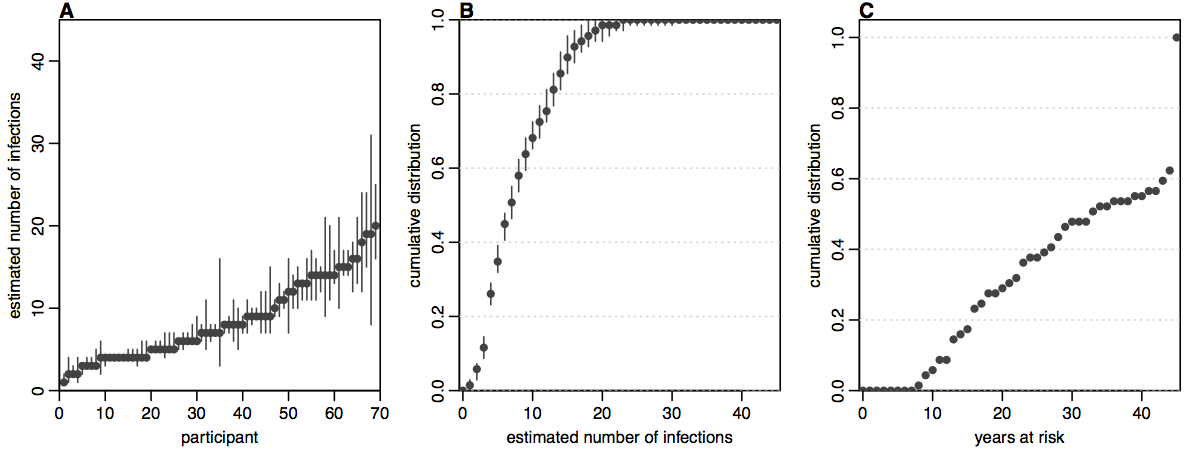

Supplement: S12 Fig — (A) Distribution of estimated number of infections for each participant, with median and 95% credible interval shown. (B) Cumulative distribution of estimated infections for study participants, with median and 95% CrI shown. Values calculated by sampling from the posterior cumulative distribution of total infections for participants. (C) Cumulative distribution of number of years at risk for A/H3N2 infection for study participants (i.e., number of years alive in the period post 1968). HI, haemagglutination inhibition. (TIFF) [file pbio.2004974.s012.tiff]

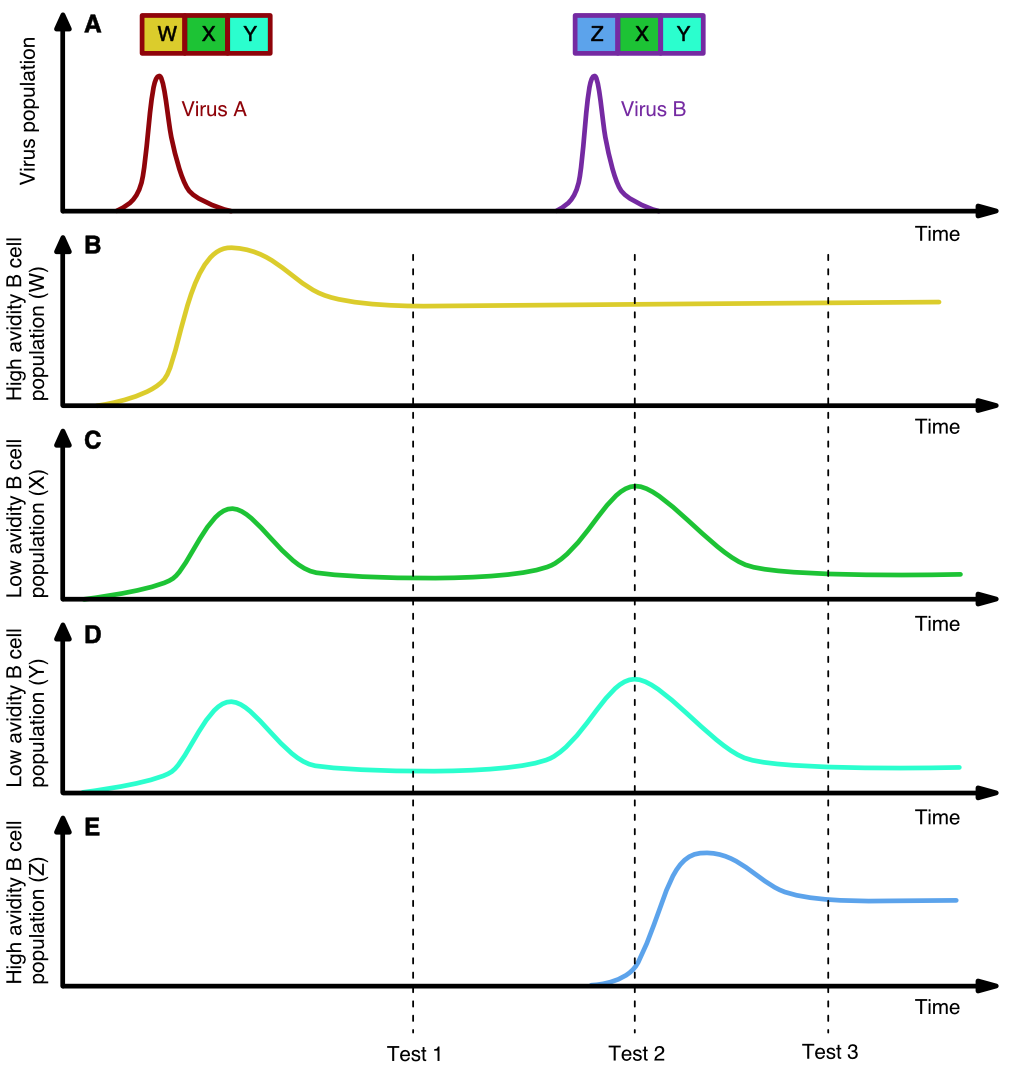

Supplement: S13 Fig — (A) In this simple illustration, each virus has 3 epitopes that can be targeted by monoclonal antibodies. The first infection, with virus A, stimulates distinct populations of memory B cells within the host that produce (B) antibodies with high avidity to epitope 1 and (C–D) antibodies with lower avidity to epitopes 2 and 3. After clearance of virus, these B cell populations decline to an equilibrium level. A serological sample tested at this point (Test 1) would exhibit a long-term response specific to virus A only. Upon secondary infection with virus B—which has epitopes 2 and 3 but with a new epitope 4 in place of epitope 1—the lower-avidity B cell populations are activated, along with (E) a newly stimulated B cell population that has high avidity to epitope 4. However, the virus population is neutralised before these B cells reach the level of earlier B cell populations, which produce the ‘antigenic seniority’ effect. Following the secondary infection, the host would exhibit raised levels of antibodies against epitopes 2 and 3 and hence produce a response even against viruses with only one of these epitopes. This results in a short-lived, broadly cross-reactive serological response (Test 2), which wanes to leave a narrower long-term response (Test 3). (TIFF) [file pbio.2004974.s013.tiff]

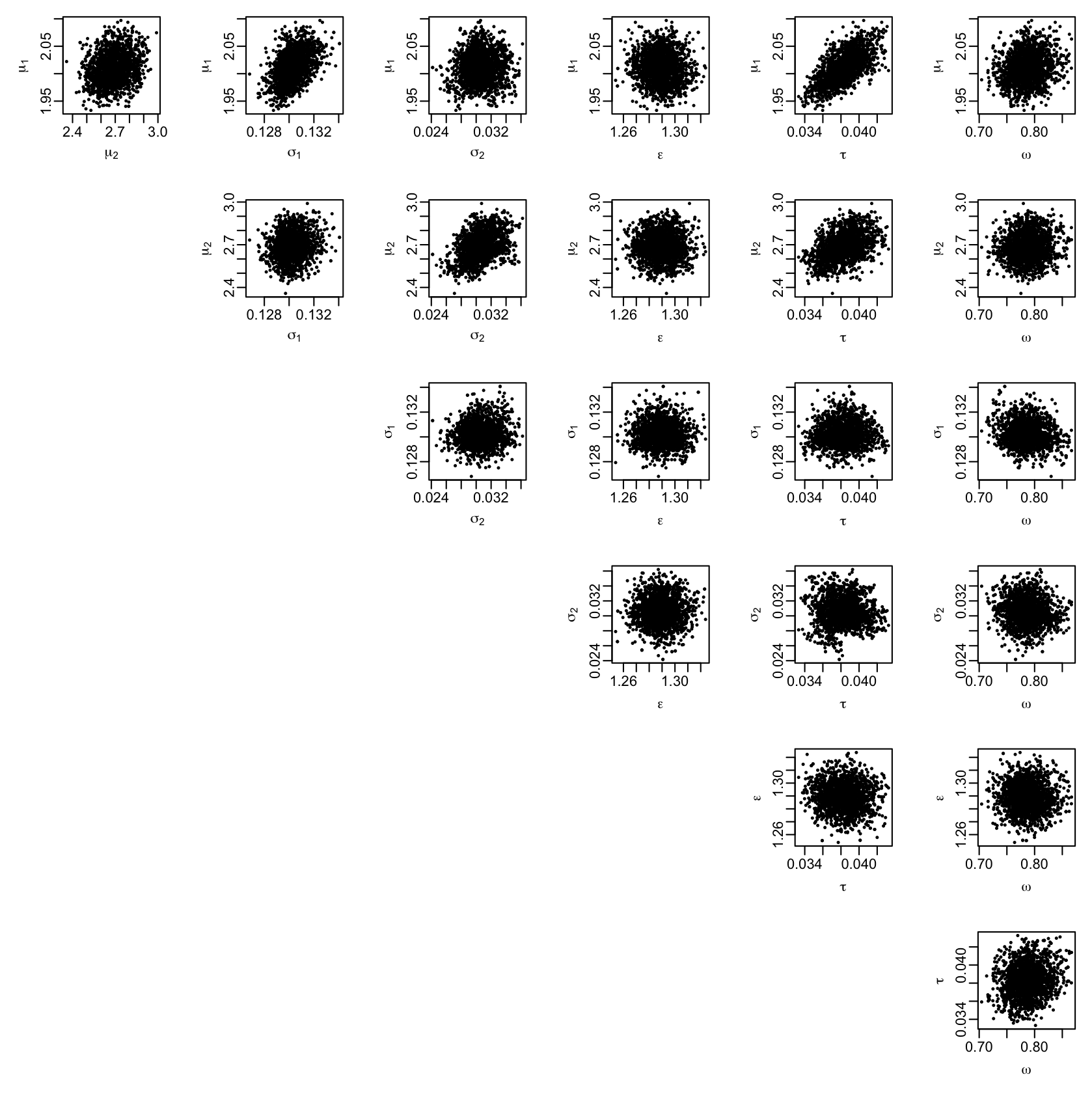

Supplement: S14 Fig — Pairwise plots show 1,000 MCMC samples from the full joint posterior distribution. HI, haemagglutination inhibition; MCMC, Markov chain Monte Carlo. (TIFF) [file pbio.2004974.s014.tiff]

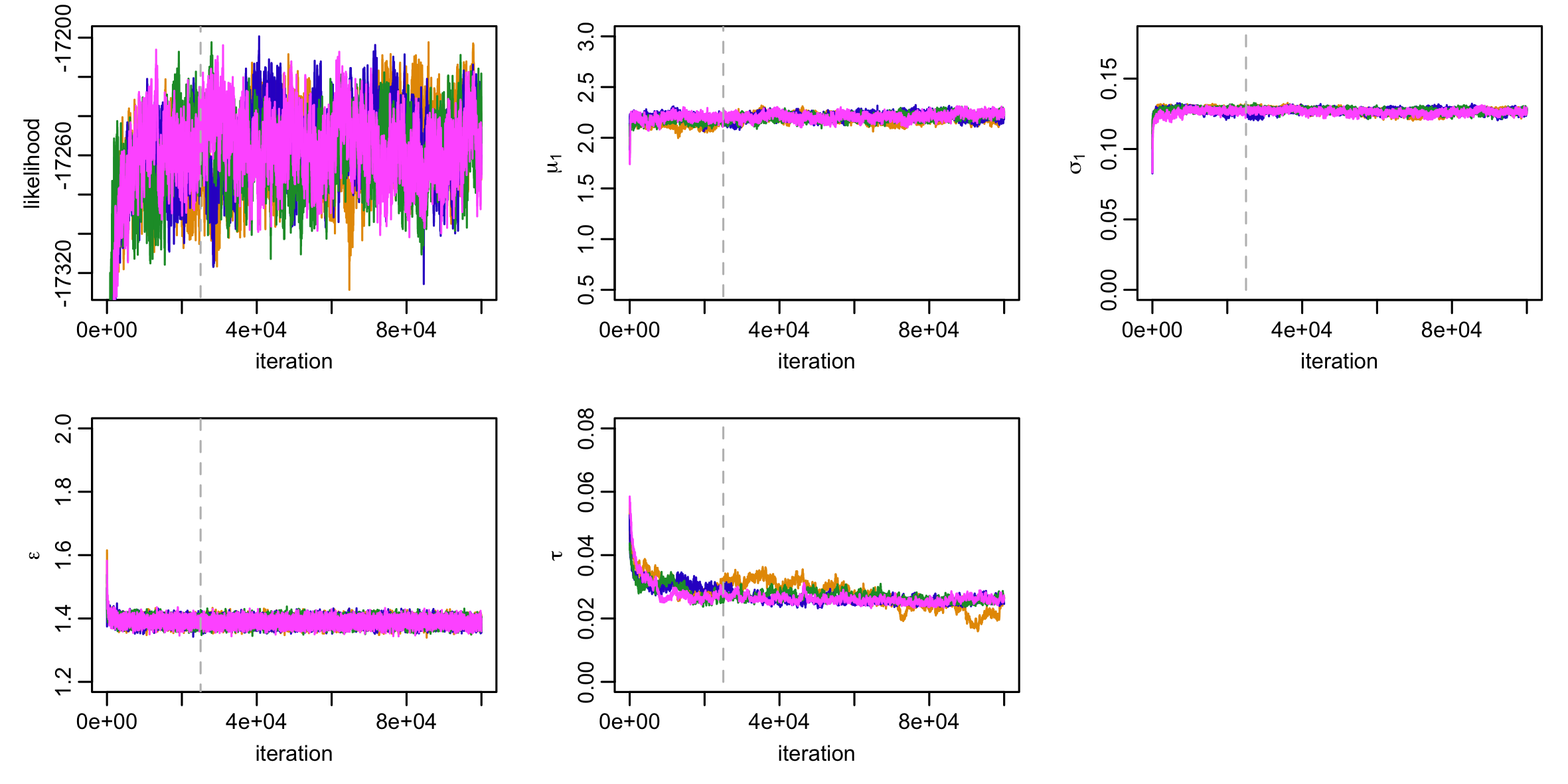

Supplement: S15 Fig — Dashed line shows burn-in period. HI, haemagglutination inhibition; MCMC, Markov chain Monte Carlo. (TIFF) [file pbio.2004974.s015.tiff]

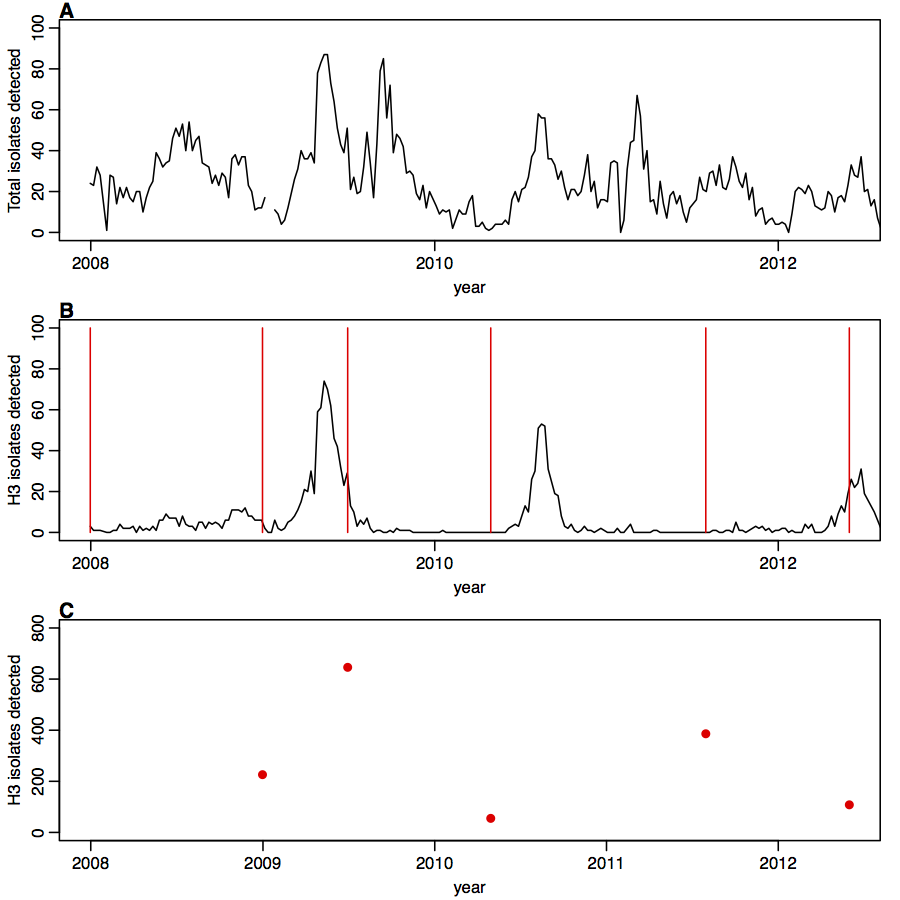

Supplement: S16 Fig — (A) All influenza isolates reported [50]. (B) A/H3N2 isolates. Red lines show times of serological sampling. (C) Cumulative isolates in each period. (TIFF) [file pbio.2004974.s016.tiff]
