# Supplementary material for: Timescales of influenza A/H3N2 antibody dynamics
Source: PLoS Biol. 2018 Aug 20;16(8):e2004974. doi: 10.1371/journal.pbio.2004974 (PMC6117086; doi:10.1371/journal.pbio.2004974)
Supplement: S1 Table — Median estimate shown, with 95% credible interval in parentheses. ESS for each parameter is also shown, to indicate the extent of autocorrelation in MCMC sampling. ESS, effective sample size; MCMC, Markov chain Monte Carlo. (PDF) [file pbio.2004974.s017.pdf]

Parameter	China (2009, microneut)	ESS	China (2009, HI)	ESS	Vietnam (2007–2012, HI)	ESS
Long-term boost (μ_1)	1.38 (1.14-1.66)	494	0.972 (0.854-1.13)	662	2.02 (1.96-2.08)	824
Short-term boost (μ_2)	–	–	–	–	2.69 (2.5-2.88)	740
Long-term cross-reaction (σ_1)	0.13 (0.106-0.146)	832	0.099 (0.0838-0.114)	826	0.13 (0.128-0.132)	2390
Short-term cross-reaction (σ_2)	–	–	–	–	0.0307 (0.0263-0.0348)	294
Observation error (ε)	1.69 (1.56-1.82)	1660	1.5 (1.41-1.59)	3800	1.29 (1.27-1.31)	9310
Antigenic seniority (τ)	0.0202 (0.0153-0.027)	316	0.0156 (0.0118-0.0205)	332	0.0385 (0.0353-0.0417)	325
Waning (ω)	–	–	–	–	0.787 (0.738-0.837)	1150
